# P2X7 a new therapeutic target to block vesicle-dependent metastasis in colon carcinoma: Role of the A2A/CD39/CD73 axis

**DOI:** 10.1038/s41419-025-07897-2

**Published:** 2025-08-04

**Authors:** Anna Pegoraro, Elena De Marchi, Luigia Ruo, Michele Zanoni, Sofia Chioccioli, Giovanna Caderni, Letizia Alfieri, Marianna Grignolo, Paola Ulivi, Alessandro Passardi, Graziana Gallo, Luca Antonioli, Francesco Di Virgilio, Elena Adinolfi

**Affiliations:** 1 Department of Medical Sciences, Section of Experimental Medicine, University of Ferrara, Ferrara, Italy; 2 Biosciences Laboratory, IRCCS Istituto Romagnolo per lo Studio dei Tumori (IRST) “Dino Amadori”, Meldola, Italy; 3 NEUROFARBA Department, Pharmacology and Toxicology Section, University of Florence, Florence, Italy; 4 Department of Medical Oncology, IRCCS Istituto Romagnolo per lo Studio dei Tumori (IRST) “Dino Amadori”, Meldola, Italy; 5 Operative Unit of Pathologic Anatomy, Azienda USL della Romagna, “Maurizio Bufalini” Hospital, Cesena, Italy; 6 Department of Clinical and Experimental Medicine, University of Pisa, Pisa, Italy

**Keywords:** Metastasis, Cancer microenvironment, Colon cancer, Ion channel signalling, Extracellular signalling molecules

## Abstract

Extracellular vesicle-driven cancer metastasis represents a therapeutic challenge due to the lack of effective blocking drugs. Our study shows that activation of the P2X7 receptor on colon carcinoma cells causes the release of vesicles carrying CD39 and CD73 ectonucleotidases. These vesicles increase ATP and adenosine levels and, when in vivo administered, significantly enhance colon carcinoma metastasis and circulating levels of vesicles after fourteen days from their injection. Blocking P2X7 prevents vesicular release and substantially reduces vesicle-mediated tumor spreading, positioning P2X7 as a promising therapeutic target for inhibiting extracellular vesicle-mediated dissemination in colon cancer. Additionally, these vesicles upregulate the expression of P2X7 and A2A receptors within the metastatic niche. Antagonists of P2X7 and A2A used alone or in combination effectively inhibit tumor growth in vivo, decreasing metastasis engraftment and IL-17 and IL-23 release. Interestingly, the levels of both cytokines were also reduced by combined P2X7 and A2A blockade in non-tumor-bearing mice. Moreover, P2X7 and A2A upraise in metastatic and *APC*-mutated colon carcinoma patients and in *Apc*-disrupted rats. Our findings shed light on the crosstalk of P2X7/CD73/CD39 and A2A in colon cancer metastasis. We propose a novel mechanism facilitating metastatic dissemination and an innovative therapeutic strategy to target receptor signaling and vesicular release.

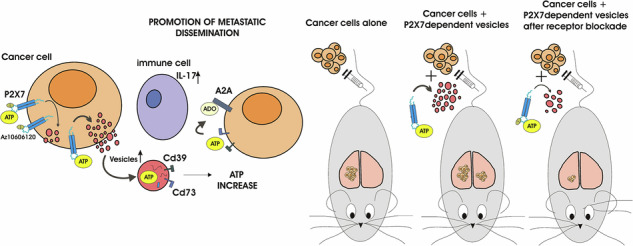

## Introduction

The tumor microenvironment (TME) is rich in extracellular ATP (eATP) and its hydrolytic derivative adenosine, which promotes pro and anti-tumor pathways acting on tumor cells and immune infiltrates [1–3]. The best-characterized receptor for eATP in cancer is P2X7, which is expressed by both immune and tumor cells, promoting cancer growth [4], neovascularization [5], dissemination [6], and release of its ligand ATP [7], as well as activating tumor-eradicating immune responses [8–10]. ATP and adenosine levels are strictly intertwined as, in the extracellular milieu, adenosine is produced from ATP by phosphates group loss mediated by the ectonucleotidases CD39 and CD73 [10]. Interestingly, these enzymes and the primary adenosine receptor expressed in cancer (A2A) are emerging therapeutic targets in clinical trials designed to relieve immunosuppression and circumvent resistance to tumor immunotherapy [3, 11]. In addition, in the TME, A2A promotes VEGF secretion, tumor cell proliferation, and dissemination [12, 13]. Data emerging from our group and others strongly support the existence of crosstalk between the purinergic and adenosinergic systems in promoting cancer. For example, tumors growing in *P2rx7* null mice overexpress CD73, CD39, and A2A in either immune-infiltrating or tumor cells [7, 14]. Moreover, the mechanisms leading to the effectiveness of anti-CD39 antibodies as immune system-reactivating drugs depend on P2X7 [15–17], highlighting the close link between key members of the purinergic system in the TME. Taken together, all these findings prompted researchers in the field to collectively designate P2X7, CD39, CD73, and A2A as purinergic checkpoints [18].

Extracellular vesicles, including exosomes, microvesicles, and other emerging populations such as migrasomes or mitovesicles, have been identified as excellent tumor biomarkers and mediators of cancer transformation and progression, influencing metastatic dissemination [19, 20]. Cancer cells released from the primary tumor can engraft only in a favorable microenvironment known as the metastatic niche. The release of vesicles is a mechanism by which cancer cells influence the composition of distant extracellular microenvironments, causing the so-called metastatic niche preconditioning effect: reprogramming the secondary organ sites to favor tumor cell engraftment and, therefore, metastasis formation [21]. However, although extracellular vesicles blockade holds promise as a therapeutic approach to prevent metastasis, no drugs able to interfere with this process are actually available on the market, probably because most of the targets identified so far are intracellular [22].

The P2X7 receptor was long known for its ability to trigger the release of extracellular vesicles containing IL-1β, IL-18, and other cytokines from immune and nervous system cells [23]. We have recently demonstrated that melanoma cells can also release exosomes and microvesicles upon P2X7 stimulation and that these particles are characterized by miRNA content that is profoundly different from spontaneously released vesicles [6]. Interestingly, vesicles released following P2X7 activation also contain ATP [6, 24, 25], thus suggesting that they might shape purinergic signaling in the TME. Indeed, it has been shown that extracellular vesicles can carry the ectonucleotidases CD39 and CD73 and degrade ATP into immunosuppressive adenosine, supporting cancer growth [26]. However, an in-depth investigation was missing linking the P2X7 /CD39/CD73 /A2A axis with extracellular vesicle release and the ability of these particles to influence ATP and adenosine levels in the TME and promote metastasis. Our study investigates P2X7-dependent vesicle secretion as a possible cause of colorectal cancer (CRC) metastasis due to changes in purinergic signaling and its antagonism as a new therapeutic approach to avoid cancer dissemination.

CRC is one of the most common cancers and the second leading cause of cancer-death worldwide. CRC risk factors include poor diet, physical inactivity, alcohol intake, and inflammatory bowel diseases [27, 28]. The survival rates of patients with early-stage disease are increasing because of the efficacy of surgery, which is often combined with adjuvant therapy. However, late-stage metastatic CRC remains a clinical challenge [29]. A bad prognosis due to increased metastatic dissemination is frequently due to mutations in the adenomatous polyposis coli (*APC*) gene associated with familiar forms of CRC but also often found in sporadic carcinomas [30].

Although P2X7, CD39, CD73, and A2A have been separately associated with CRC development and progression [31–35] a systematic analysis of their crosstalk in metastatic spreading was missing.

## Results

### P2X7 blockade reduces the release of extracellular vesicles from colon carcinoma cells

**Fig. 1:**
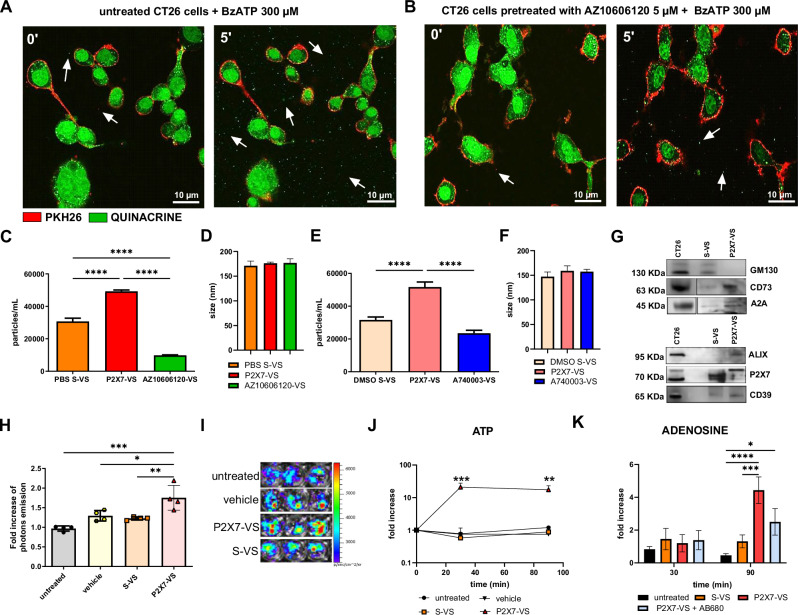
Colon carcinoma cells release vesicles upon P2X7 activation. **A** CT26 CRC cells were stained with PKH26GL (red) and quinacrine (green) fluorescent dyes. Images were acquired using confocal microscopy at time 0 and after 5 min following P2X7 activation with 300 µM BzATP and are extrapolated from a 30-min time course (see supplementary videos 1 and 3). **B** CT26 cells were pre-treated with P2X7 antagonist AZ10606120 (5 µM) for 10 min before application of BzATP. **C** Number of vesicles released in 30 min from CT26 cells in PBS vehicle (PBS S-VS), following stimulation with P2X7 agonist ATP (P2X7-VS) or 10 min pretreatment with P2X7 antagonist AZ10606120 followed by stimulation with 3 mM ATP (AZ-VS) (*n* = 5). **D** Size of PBS S-VS, P2X7-VS, and AZ-VS (*n* = 5). **E** Number of vesicles released in 30 min from CT26 cells in DMSO vehicle (DMSO S-VS), following stimulation with P2X7 agonist ATP (P2X7-VS) or 10 min pretreatment with P2X7 antagonist A740003 followed by stimulation with 3 mM ATP (A74-VS) (*n* = 7). **F** Size of DMSO- S-VS, P2X7-VS, and A74-VS (*n* = 7). **G** Western blot for GM130, Alix, P2X7, CD39, CD73, and A2A in CT26 cells, S-VS and P2X7-VS. Pericellular ATP was measured with the pmeLUC probe expressed on the cell surface of untreated CT26 cells or after 5 min of exposure to PBS vehicle, S-VS, and P2X7-VS (*n* = 4). **H** Quantification of luminescence changes was expressed as a fold increase on time 0. **I** Representative images of photon emissions. Changes in ATP **J** concentration increase on time 0 in the supernatants of CT26 cells, untreated or treated with PBS vehicle, S-VS, or P2X7-VS, measured with a luciferin/luciferase assay (*n* = 3). Changes in adenosine **K** concentration increase on time 0 in the supernatants of CT26 cells untreated or treated with PBS vehicle, S-VS, P2X7-VS, or P2X7-VS plus 5uM CD73 inhibitor AB680 (*n* = 5). *p < 0.05, **p < 0.001, ***p < 0,0001, ****p < 0.00001.

We recently demonstrated the release of extracellular vesicles and exosomes following P2X7 stimulation in melanoma cells and how their miRNA content affects cancer cell migration [6]. Therefore, we investigated whether a similar mechanism can be activated by the P2X7 receptor in colon carcinoma cells. At this aim, we selected CT26 and HCT116 colon carcinoma cell lines that both express a fully functional P2X7 receptor (Supplementary Figs. 1 and 2). Figure 1 shows that stimulation of P2X7 with its most potent agonist, BZ-ATP, causes the release of particles detectable by confocal microscopy in CT26 colon carcinoma cells (Fig. 1A, Supplementary video 1). This phenomenon can also be activated in HCT116 cells (Supplementary video 2). Interestingly, treatment with the P2X7 negative allosteric modulator AZ10606120 reduced particle release, as shown by confocal (Fig. 1B, Supplementary video 3) and particle detection analysis (Fig. 1C). Blockade with another receptor antagonist, A740003, which has a distinct chemical structure from AZ10606120, caused a comparable reduction in particle release, thus confirming the efficacy of receptor antagonism in decreasing vesicular release from cancer cells (Fig. 1E). The mean diameter of the detected particles, measured using nanosight technology, was approximately 150–200 nm and did not vary upon P2X7 stimulation or antagonism (Fig. 1D,F). Western Blot analysis of the vesicular content showed that particles released following ATP stimulation lost GM130 while gaining Alix staining compared to spontaneously released particles (Fig. 1G and Supplementary Fig. 2), suggesting P2X7 activation-dependent changes in the vesicular nature and content. Interestingly, these particles also expressed P2X7, CD39, CD73, and A2A, indicating that they may regulate the purinergic/adenosinergic axis in the TME (Fig. 1G and Supplementary Fig. 2). The molecular size of P2X7 detected by immunoblot in P2X7-VS seemed to be compatible with a glycosylated form of the receptor of approximately 80-90 KDa present also in CT26 cellular lysates. Moreover, particles caused an increase in pericellular ATP levels, which were more abundant when the vesicles were collected following P2X7 stimulation (Fig. 1H, I). These measurements were performed using the pmeLUC probe, which allows the measurement of extracellular ATP on the plasma membrane of cancer cells expressing it [24, 36]. These data were confirmed by measuring free ATP in the supernatant using a classical luciferin/luciferase assay (Fig. 1J). Interestingly, adenosine levels in the cell supernatants were also increased by treatment with particles isolated following P2X7 stimulation and showed a tendency towards reduction following CD73 blockade (Fig. 1K).

### In vivo administration of P2X7-released vesicles enhances colon carcinoma cell dissemination

**Fig. 2:**
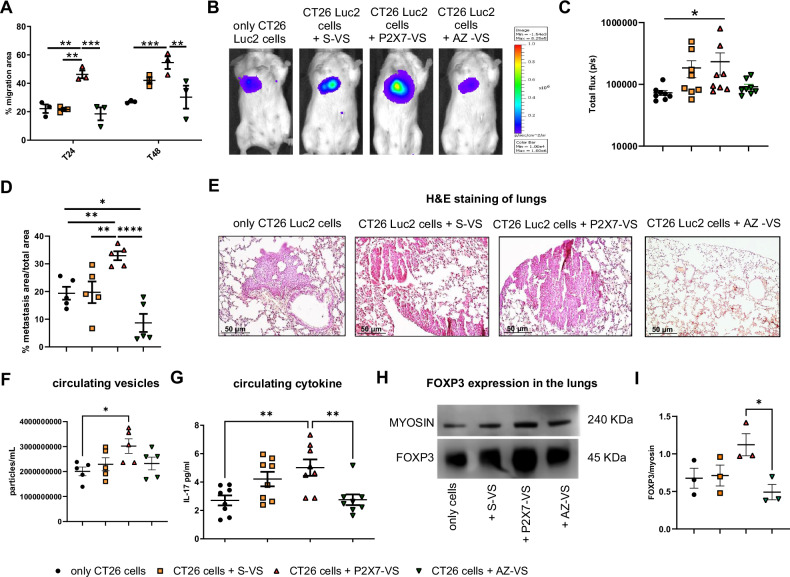
Vesicles released from CRC cells upon P2X7 stimulation increase cell dissemination and metastasis formation. **A** Quantification of the migration capacity of CT26 cells alone or incubated with S-VS, P2X7-VS, and AZ-VS. The migration ability is expressed as the percentage of the area covered by cells evaluated 24 and 48 h after the scratch formation (*n* = 3). Mice were intravenously injected with CT26 Luc2 cells alone or pre-treated with 4 × 10^9^ S-VS, P2X7-VS, or AZ-VS. The vesicles were obtained from batches of cells different from those injected; see materials and methods. **B** Representative images of luminescence emission on day 14 from the inoculum. **C** Quantification of photon emission, expressed as a total flux, at day 14 (*n* = 8). **D** The area of the lung covered by metastasis is expressed as a percentage of the total lung area (*n* = 5). **E** Representative hematoxylin/eosin staining of mouse lungs. **F** Number of particles measured in the mice’s plasma at day 14 (*n* = 5). Systemic level of the proinflammatory cytokines IL-17 (**G**, *n* = 8). **H** Representative Western blot of FOXP3 expression in mice’ lungs. **I** Densitometric analysis of FOXP3. Data were normalized on myosin (*n* = 3). *p < 0.05, **p < 0.001, ***p < 0,0001, ****p < 0.00001.

**Fig. 3:**
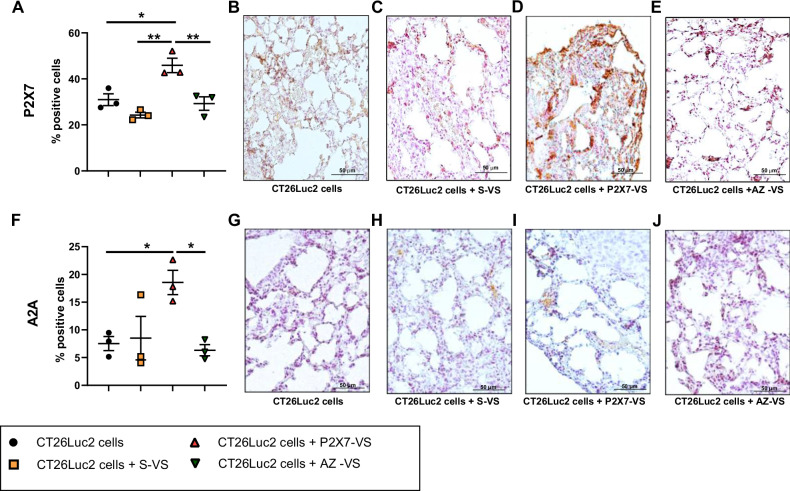
Vesicles released from CRC cells upon P2X7 stimulation increase the expression of P2X7 and A2A in the metastatic milieu. Percentage of lung cells positive to P2X7 **A** and A2A **F** staining in mice injected intravenously with only CT26 Luc2 cells or CT26 Luc2 pre-treated with S-VS, P2X7-VS, and AZ-VS (*n* = 3). Representative immunohistochemistry images of mice injected intravenously with CT26 Luc2 alone **B**,**G** or pre-treated with S-VS **C**,**H**, P2X7-VS **D**,**I**, or AZ-VS **E**,**J**. *p < 0.05, **p < 0.001.

To understand whether extracellular vesicles released upon P2X7 activation could play a role in colon carcinoma metastatic processes, we analyzed the effect of their administration on CT26 cell mobility with a scratch test assay (Fig. 2A). These experiments showed that only particles released upon P2X7 stimulation (P2X7-VS) and not those spontaneously released (S-VS) were able to increase colon carcinoma cell spreading in vitro. Moreover, if vesicles were collected from cells exposed to P2X7 antagonist together with its agonist (AZ-VS), the spreading advantage was lost (Fig. 2A). Similar results were obtained in vivo in a metastatic model obtained by injecting CT26 cells, expressing an intracellular luciferase (CT26 Luc2) in the caudal vein of BALB/c syngeneic mice. Pretreatment of CT26 Luc2 with vesicles released upon P2X7 activation significantly increased cancer cell spreading in the lungs and metastasis formation (Fig. 2B–E). Moreover, P2X7-VS pre-administration to CRC cells induced an increased production of particles measured systemically in mice 14 days after their inoculation, suggesting a priming effect leading to additional vesicular release (Fig. 2F). Activation of the P2X7 receptor preceding EV collection was central in EV-linked metastatic spreading and in vivo particles production as receptor blockade in this phase prevented these phenomena (see AZ-VS controls Fig. 2B–F). Interestingly, P2X7-VS also increased systemic levels of the proinflammatory tumor-promoting cytokine IL-17 (Fig. 2G). The expression of Foxp3, a nuclear factor associated with Treg cells, including those derived from Th17 [37], was increased by P2X7-VS as compared to AZ-VS (Fig. 2H, I). Co-administration of microparticles released upon P2X7 stimulation not only increased cancer spreading (Fig. 2) but also tissue expression of both P2X7 (Fig. 3A–E) and A2A (Fig. 3F–J).

### P2X7 A2A combined antagonism reduces colon carcinoma growth, dissemination, and circulating IL-17 and IL-23 in a syngeneic mice model

**Fig. 4:**
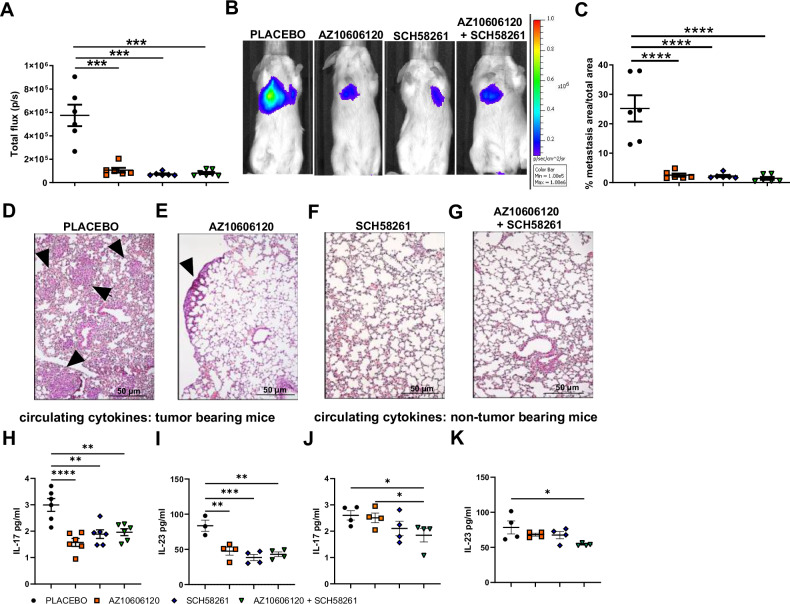
P2X7 and A2A blockade reduces in vivo dissemination of CRC cells and systemic levels of IL-17 and IL-23. Mice were injected intravenously with CT26 Luc2 cells and treated intraperitoneally with placebo, the P2X7 antagonist AZ10606120 (2 mg/Kg), the A2A antagonist SCH58261 (1 mg/Kg), or both drugs every three days. **A** Quantification of photon emission, expressed as total flux (p/s), on day 14 from the inoculum (*n* = 6) **B** Representative images of photon emission at day 14 from the inoculum. **C** Area of metastasis expressed as a percentage of total lung area (*n* = 6). Representative hematoxylin/eosin staining of lungs from mice treated with **D** placebo, **E** the P2X7 antagonist AZ10606120 (2 mg/Kg), **F** the A2A antagonist SCH58261 (1 mg/Kg), and **G** combination of the two drugs. Black arrows indicate metastatic lesions. Systemic levels of **H** IL-17 (*n* = 6) and **I** IL-23 (*n* = 4) were measured in the plasma of tumor-bearing mice. Systemic levels of **J** IL-17 (*n* = 4) and **K** IL-23 (*n* = 4) measured in the plasma of mice not injected with tumor cells but treated only with placebo, AZ10606120 (2 mg/Kg), SCH58261 (1 mg/Kg), or both compounds.*p < 0.05, **p < 0.001, ***p < 0.0001, ****p < 0.00001.

**Fig. 5:**
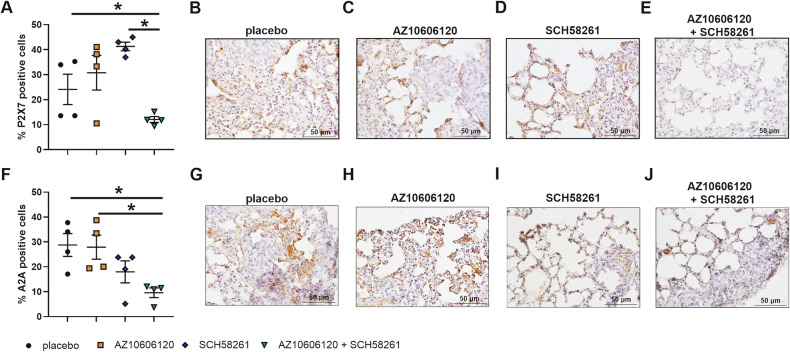
P2X7 and A2A combined antagonism decreases P2X7 and A2A expression in the metastatic niche. **A–J** BALB/c mice were injected intravenously with CT26 Luc2 cells and treated intraperitoneally with placebo, AZ10606120 (2 mg/Kg), SCH58261 (1 mg/Kg), or a combination of both drugs. Percentage of lung cells positive for P2X7 **A** and A2A **F** staining (*n* = 4). Representative immunohistochemistry images of mice treated with placebo **B**, **G**, P2X7 antagonist AZ10606120 (C, H), A2A antagonist SCH58261 **D**,**I**, or both drugs **E**,**J**. *p < 0.05.

To investigate the possible role of adenosine and the A2A receptor in P2X7-dependent metastatic dissemination, we tested the effects of P2X7 and A2A double blockade in vivo. P2X7 and A2A antagonism alone or in combination proved effective in reducing the spread of CT26 Luc2 in our metastatic model (Fig. 4A–G). P2X7 antagonist AZ10606120 and A2A antagonist SCH58261 were i.p. administered alone or in combination every three days, starting on the day of CRC i.v. inoculum (Fig. 4). The significant reduction in metastatic spreading and engraftment observed with either P2X7 or A2A blockade suggests that both receptors favor CRC dissemination. Of interest, P2X7 and A2A blockade also reduced systemic levels of IL-17 (Fig. 4H) and of IL-23 (Fig. 4I) in tumor-bearing mice. AZ10606120 and SCH58261 combination decreased the secretion of both cytokines, also in non-tumor-bearing mice, suggesting a role of the P2X7-A2A axis in the production of these cytokines, also in physiological conditions (Fig. 4J–K). Moreover, only the combined antagonism of both receptors reduced the expression of P2X7 and A2A in the lungs of the metastasis-bearing mice (Fig. 5 A–J).

**Fig. 6:**
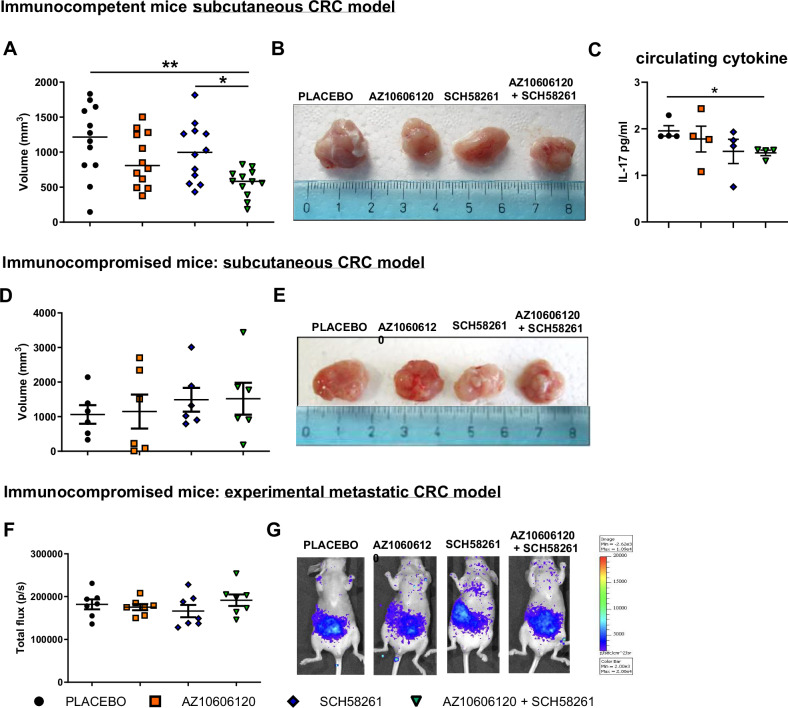
Double antagonism of P2X7 and A2A significantly reduces tumor growth in a syngeneic subcutaneous CRC model but is not efficacious in immunocompromised *nude* mice. BALB/c mice were subcutaneously injected with CT26 and treated with placebo, AZ10606120 (2 mg/Kg), SCH58261 (1 mg/Kg) or both drugs (*n* = 12). **A** Ex vivo tumor volume on day 14, and **B** representative images of tumors. **C** Systemic levels of the proinflammatory cytokine IL-17 (*n* = 4). *nude/nude* mice were subcutaneously injected with HCT116 cells and treated i.p. with placebo, AZ10606120 (2 mg/Kg), SCH58261 (1 mg/Kg) or both drugs. **D** Ex vivo tumor volume at day 14 from the inoculum (*n* = 6). **E** Representative tumor image. **F** HCT116 Luc2 were injected into the tail vein of *nude/nude* mice and treated with placebo, AZ10606120 (2 mg/Kg), SCH58261 (1 mg/Kg) or both drugs (*n* = 7). Photon emission on day 14 total flux (p/s). **G** Image representative of data shown in **F**. *p < 0.05, **p < 0.001.

Interestingly, when CT26 CRC cells were administered in BALB/c mice subcutaneously, only co-administration of both P2X7 and A2A antagonist was effective in reducing cancer growth (Fig. 6A, B) and the levels of IL-17 (Fig. 6C). Finally, when we performed similar experiments with HCT116 human CRC cells in *nude* mice, we did not measure any effect of P2X7 and A2A blockade alone or in combination, either in the subcutaneous (Fig. 6D, E) or intravenous model (Fig. 6F, G), further suggesting strong involvement of the immune response in the mechanism of action of the drugs.

### *P2X7*, *CD39*, *CD73*, and *A2A* are upregulated in metastatic colon carcinoma patients

**Fig. 7:**
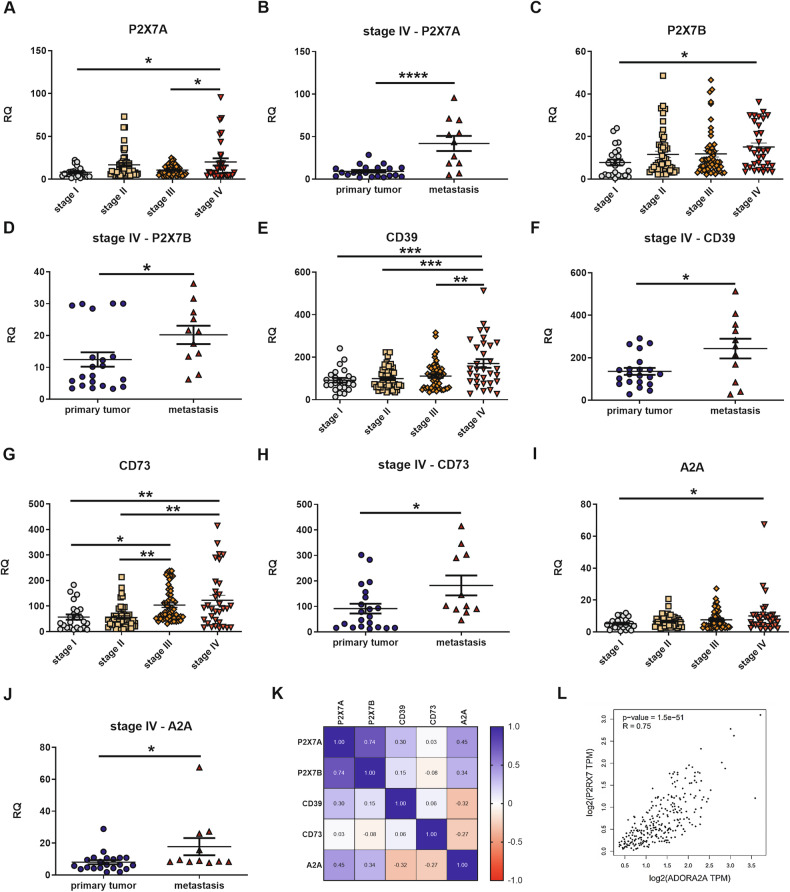
*P2X7*, *CD39*, *CD73*, and *A2A* expression significantly increased in the advanced stages of CRC. The mRNA expression of *P2X7A* **A**, **B**, *P2X7B* **C**, **D**, *CD39* **E**, **F**, *CD73* **G**, **H**, and *A2A* **I**, **J** was evaluated in the cDNAs of 158 patients with CRC subdivided into stage I (*n* = 24), stage II (*n* = 50), stage III (*n* = 52), and stage IV (*n* = 32) which comprised 11 samples derived from metastases in organs other than the colon. **K** Spearman’s correlation coefficient among *P2X7A, P2X7B, CD39, CD73*, and *A2A* was evaluated in CRC metastatic patients. **L** Spearman’s correlation coefficient was evaluated between *P2X7* and *A2A* expression in colon adenocarcinoma samples obtained from the Cancer Genome Atlas database. *p < 0.05, **p < 0.01, ***p < 0.001. ****p < 0.0001.

To understand whether our findings on the role of the purinergic adenosinergic axis could also be translated to patients, we analyzed the expression of *P2X7*, *CD39*, *CD73*, and *A2A* in an array of 158 CRC specimens (Fig. 7). Our analysis showed that upregulation of both P2X7 human isoforms, *P2X7A* and *P2X7B* [38, 39] are associated with stage IV CRC (Fig. 7A, C), a subset of patients characterized by a bad prognosis and metastatic dissemination. Similar results were obtained for *CD39* (Fig. 7E), *CD73* (Fig. 7G), and *A2A* (Fig. 7I). In our tested samples, the mRNA that best increased according to the CRC stage was *CD73* (Fig. 7G). Interestingly, when we further analyzed data from stage IV CRC samples, subdividing them into those obtained from primary tumors versus metastatic specimens, we found upregulation of all purinergic checkpoints in secondary metastatic forms (Fig. 7B, D, F, H, J). In these samples, we also reported a positive correlation between the expression levels of *P2X7A* and *P2X7B* (Spearman’s coefficient 0,74), *P2X7A* and *A2A* (Spearman’s coefficient 0,45), *P2X7B* and *A2A* (Spearman’s coefficient 0,34) (Fig. 7K). A moderate positive correlation between *P2X7* and *A2A* was confirmed by the analysis of CRC samples from the Atlas database (Fig. 7L). However, it was impossible to distinguish between *P2X7A* and B isoforms in this case.

### *APC* mutation in patient specimens and the PIRC rat correlates with *P2X7* and *A2A* overexpression

**Fig. 8:**
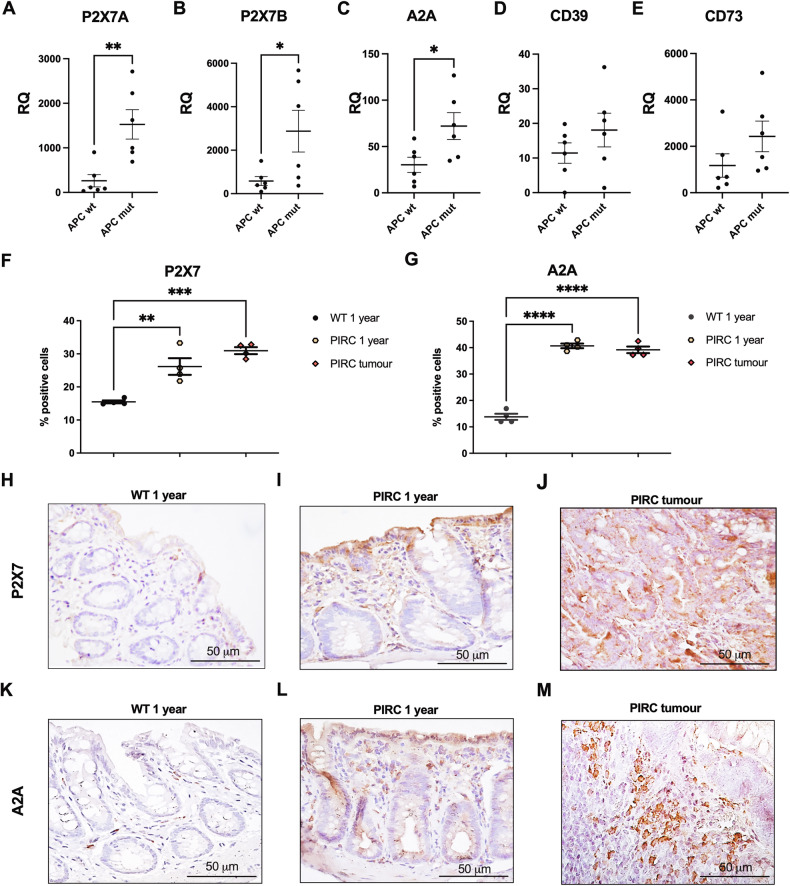
P2X7 and A2A expression is enhanced in PIRC rats and *APC*-mutated CRC patients. mRNA expression of **A** *P2X7A*, **B** *P2X7B*, **C** *A2A*, **D** *CD39*, and **E** *CD73* in CRC patients subdivided into *APC* WT and *APC* mutated groups (*n* = 6). Percentage of cells positive for P2X7 **F** and A2A **G** in the colons of WT and PIRC rats and PIRC tumors (*n* = 4). Representative images of immunohistochemical staining for P2X7 and A2A in the colon of WT 1-year rats **H, K** and in the normal colon **I, L** and the tumor mass **J, M** of 1-year PIRC rats. *p < 0.05, **p < 0.01, ***p < 0.001, ****p < 0.0001.

*APC* oncogene mutational status is often associated with bad prognosis and metastatic dissemination in CRC patients. Therefore, to further corroborate our data, we also analyzed *P2X7A*, *P2X7B*, *CD39*, *CD73*, and *A2A* expression in mRNA extracted from human CRC samples coming from *APC* WT versus *APC* mutated tumors (Fig. 8A–E). Interestingly, *P2X7A*, *P2X7B*, and *A2A* mRNA were increased in *APC* mutated tumors. In the same samples, the levels of *CD39* and *CD73* also showed a tendency to be upregulated in *APC*-mutated patients (Fig. 8D, E). To substantiate the association between P2X7 and A2A to *APC* mutational status, we took advantage of a murine genetic model of intestinal tumorigenesis: PIRC rats carrying an *Apc* mutation. These rats spontaneously develop tumors in the colon that faithfully reproduce familiar and sporadic CRC in humans [40, 41]. Immunohistochemistry demonstrated an upregulation of P2X7 and A2A in both the colon mucosa and colon tumors of PIRC rats as compared to the colon mucosa of WT rats (Fig. 8F–M). Interestingly, this upregulation appeared to involve both cancer and immune cells (Fig. 8J, M). In contrast, CD39 and CD73 levels were not altered in PIRC rats (Supplementary Fig. 3).

## Discussion

CRC is the third most common tumor, accounting for a high number of cancer-related deaths worldwide [42]. Despite recent advancements in survival expectations owing to hepatic metastasis resection, personalized therapy, the use of biologics, and immunotherapy, the metastatic forms of CRC remain a clinical challenge [43, 44]. These premises formed the basis of our study, which aimed to associate vesicular release triggered by extracellular ATP (eATP) and the purinergic axis formed by P2X7/CD39/CD73 and A2A with CRC metastatic dissemination. Indeed, extracellular vesicles play important roles in preconditioning the metastatic niche, transforming it into a hospitable, tumor-friendly milieu that supports the engraftment and growth of cancer cells [45].

Extracellular ATP and its degradation product, adenosine, have been associated with CRC development, immune escape, and dissemination [10, 31, 46]. However, an association between P2X7-dependent vesicular release and accumulation of ATP and adenosine in the TME leading to facilitation of metastasis was still missing. Our study shows that P2X7 stimulation causes the release of extracellular vesicles from CRC cells (P2X7-VS) that can promote cancer dissemination and metastasis formation in vivo (Fig.2). Although we did not characterize P2X7-VS in detail, we know that their content differs from that of particles spontaneously released from the same cells (S-VS, Fig.1). Converging evidence indicates the presence of both microvesicles and exosomes in our samples [47], including the absence of GM130 and the presence of Alix in P2X7-VS and the mean size determined by nanosight technology of 150–200 nm (Fig. 1). P2X7 antagonists can revert ATP-mediated vesicular release, as demonstrated by confocal and nanosight experiments performed with receptor-blocking drugs of different chemical natures. Administration of P2X7-VS increases both eATP and adenosine levels in CRC cell cultures. Interestingly, eATP concentration rises both in the vicinity of the plasmalemma, as measured by luciferase expressed in the outer layer of the plasma membrane (pmeLUC) [36], and in the supernatant of the cells. The increase in adenosine following P2X7-VS administration was delayed compared with that of eATP, suggesting a hydrolytic origin. Indeed, P2X7-VS carry CD39 and CD73, implying that they can also enhance the production and accumulation of adenosine in the TME, as previously described in other oncologic contexts [26], and CD73 blockade tends to reduce adenosine levels in our model (Fig. 1K). We recently demonstrated that the P2X7-VS released by melanoma cells and the miRNAs they carry can favor cancer cell migration in vitro [6], and, we confirmed that a similar mechanism is also activated in CRC. Moreover, we extended these findings to a live model of metastatic carcinogenesis, showing not only increased dissemination and engraftment in the lungs of CRC cells pre-treated with P2X7-VS and intravenously injected in mice, but that this preconditioning even enhanced the levels of particles measured in mice plasma 14 days after the cell inoculum (Fig. 2F). More importantly, antagonism of the P2X7 receptor can eliminate the metastatic advantage conferred by vesicles. The finding that blockade of P2X7-dependent vesicular release can prevent CRC dissemination is of particular relevance given that although inhibition of extracellular vesicle secretion holds promise for preventing tumor progression [48], no drugs that target vesicular secretion have been approved for human use [22, 49]. Our data show that vesicles released upon P2X7 activation were more effective than those spontaneously released by tumor cells, allowing CRC metastasis. This effect could be due to the fact that these particles carry prometastatic miRNAs [6], ATP [6, 24, 25], and mitochondria [25, 50], which can activate the metabolic activity of cells [50] and shape the immune response [51, 52]. Moreover, the high concentrations of eATP present in the TME are compatible with continuous P2X7 activation, leading to vesicle release and even increase following most traditional oncological interventions, including chemotherapy and radiotherapy [11], suggesting that P2X7-VS could favor the relapse of oncologic conditions following common treatments. Finally, several P2X7 antagonists have already been administered in phase I and II clinical trials in patients with non-oncologic diseases, including Chron’s, with little to no side effects [53]. Therefore, they can be quickly transferred to a clinical setting to prevent vesicle-mediated metastasis.

The levels of eATP and adenosine in the TME are interdependent because adenosine is generated from ATP via two ectoenzymes, CD39 and CD73, which are also known to favor cancer growth through immune suppression. Recent evidence shows that the lack or the inhibition of P2X7 alters eATP levels and modulates CD39, CD73, and adenosine receptor A2A in both immune-infiltrating and cancer cells [7, 14, 15] strongly suggesting that the manipulation of P2X7 receptor activity affects the entire purinergic/adenosinergic axis in the TME. Indeed, we demonstrated that vesicles released upon P2X7 activation enhance both extracellular ATP and adenosine levels and that they carry not only P2X7 but also CD39, CD73, and A2A, suggesting that the P2X7-VS influences the adenosinergic axis. Additionally, P2X7-VS pretreatment of disseminating tumor cells enhanced the expression of both P2X7 and A2A receptors in tumors engrafted in the lungs. To explore whether the mechanisms driving P2X7-dependent metastasis also influenced the pro-tumoral adenosinergic pathway, we investigated the effects of concurrent blockade of P2X7 and A2A receptors in vivo. P2X7 and A2A antagonists, alone or in combination, were able to reduce the metastatic spread of CT26 CRC cells in a syngeneic mouse model. Thus confirming the importance of both receptors in the promotion of CRC metastasis. Notably, double antagonism, although not causing any evident extra reduction in metastatic spreading, was the only treatment able to decrease the expression of both P2X7 and A2A in the engrafted tumors. Thus, suggesting a more efficacious action in the downmodulation of purinergic checkpoints and hinting at an addictive effect of the double antagonism on the growth of metastases following engraftment. The administration of P2X7 or A2A antagonists alone was not sufficient to mitigate subcutaneous primary tumor growth, which was nonetheless significantly reduced by combined anti-P2X7/A2A blockade. Interestingly, none of the antagonists alone or in combination was able to reduce CRC growth in the *nude* mouse model devoid of T cell-mediated responses, implicating a central role of the immune system in their mechanism of action (Fig. 6). In fact, in the fully immune-competent syngeneic model, circulating levels of IL-17 and IL-23 were also reduced by P2X7 and A2A antagonism, following a pattern similar to that observed for tumor growth and metastatic dissemination. Moreover, double blockade of the receptors diminished both IL-17 and IL-23 levels, also in mice not inoculated with tumor cells, thus suggesting that crosstalk between P2X7 and A2A will be important in the modulation of these proinflammatory cytokines in non-oncological contexts too (Fig. 4J, K). IL-17 and IL-23 are cytokines implicated in autoimmune and inflammatory bowel diseases and the maintenance of colon homeostasis [54–56], which promotes CRC development, progression, and metastasis and reduces the efficacy of immune therapy [55, 57–59]. Both P2X7 and A2A have been previously associated with Th17 cells, IL-17 and IL-23 secretion in different pathological contexts [14, 60–64]. However, to our knowledge, this is the first demonstration that blocking P2X7 and A2A, alone or in combination, can significantly reduce the levels of IL-17 and IL-23 in physiological conditions and CRC murine models. Interestingly, in the metastatic context, IL-17 levels can also be upregulated by treatment with vesicles derived from CRC cells stimulated with the P2X7 agonist ATP, suggesting that the cargo of these vesicles comprising ATP and miRNAs [6, 25] plays a role in modulating Th17 pathways. However, our data does not univocally demonstrate that the tumor promoting effects of P2X7-VS will be solely mediated by IL-17 associated pathways and further experiments performed for example with IL-17 blocking agents, will be needed to support our findings [65–67]. Moreover, pretreatment of cancer cells with vesicles released following P2X7 blockade (AZ-VS) was able to decrease the level of Foxp3, hinting at an additional mechanism by which P2X7 receptor might be influencing CRC dissemination by shaping Tregs-mediated and other immune responses leading to CRC progression [68, 69]. Given that P2X7 is a major positive regulator of the NLRP3 pathway [70], it is tempting to speculate that at list part of Foxp3 positive cells could belong to the subclass of Foxp3+ RORγt + IL17+ cells which were recently associated with NLRP3 activation in cancer [71].

To corroborate our data in a patient cohort we demonstrated that the mRNAs levels of the two main P2X7 human isoforms, P2X7A and P2X7B, were upregulated in patients with stage IV CRC. Moreover, they almost doubled when comparing expression in primary stage IV tumors versus metastatic forms. Since P2X7B was demonstrated to positively affect P2X7A activity when they are co-expressed [72], the simultaneous overexpression of both receptors isoforms points to upregulation of all P2X7-associated protumoral functions [73]. Moreover, these data further support the association of P2X7A and B with cancer progression and therapy resistance previously demonstrated for other tumor types, such as neuroblastoma [5, 74, 75], melanoma [6], osteosarcoma [76, 77], acute myeloid leukemia [78], glioblastoma [79], and prostate cancer [80]. Similar results were obtained for CD39, CD73, and A2A expression with CD73, which was also upregulated in stage III CRC samples. When performing a correlation analysis in metastatic specimens, P2X7A and P2X7B were strongly associated; however, there was also a moderate correlation between both isoforms and A2A. This correlation was confirmed by analysis of the Atlas database, which, unfortunately, does not allow for the distinction between P2X7 isoforms. In subsequent experiments, we investigated the correlation between the upregulation of P2X7 and A2A receptors and *APC* oncogene mutational status. Our findings revealed that mRNA expression of both P2X7A and P2X7B isoforms, as well as A2A, was significantly elevated in *APC*-mutated colorectal cancer patient samples compared to wild-type controls. Similar trends were observed in tumors spontaneously developing in the colonic mucosa of *Apc*-mutated PIRC rats, showing upregulation of both receptors in mucosal, cancerous and immune cells. In contrast, the expression levels of CD39 and CD73 remained relatively unchanged in both the PIRC rat model and *APC*-mutated patients. To our knowledge, this is the first demonstration of P2X7 upregulation in a tumor that spontaneously develops in an oncogene-mutated murine model and the first association in cancer patients between P2X7 isoforms and *APC* mutations. *APC* mutations were recently correlated with a poor response to immunotherapy in CRC [81], and given the role of both receptors in the cancer immune response [8, 11], it is tempting to speculate that the upregulation of P2X7 and A2A in these patients could be partly responsible for this phenomenon. Moreover, although the number of *APC*-mutated patients we tested was limited, we noticed that those were more frequently characterized by metastatic spread in organs other than the liver (supplementary Table 1) and, therefore, more challenging to treat, were also those overexpressing P2X7 and A2A suggesting that a P2X7-A2A targeting therapy might be indicated to prevent metastatic spreading in this patient population. In conclusion, this study elucidates a novel mechanism by which vesicular release modulates ATP and adenosine levels in cancer, thereby promoting metastatic dissemination. Our findings present an exciting therapeutic opportunity to target tumoral vesicular release, potentially enhancing immune responses against cancer. This innovative approach could significantly advance the field of theranostics by providing new strategies for combating metastasis and improving patient outcomes.

## Materials/subjects and methods

### Cell cultures and transfection

CT26 murine and HCT116 human colon carcinoma cell lines (EP-CL-0071, EP-CL-0096, CliniScience, Amsterdam, Netherlands) were cultured in RPMI 1640 (Carlo Erba Reagents, Milan, Italy) and McCoy’s 5 A (Euroclone, Milan, Italy) medium, respectively. Media were supplemented with 10% heat-inactivated fetal bovine serum (FBS), 100 U/mL penicillin, and 100 mg/mL streptomycin (Euroclone). CT26 and HCT116 cells were stably transfected with intracellular Luc 2 and plasma membrane pmeLUC luciferase probes using Lipofectamine LTX (Thermo Fisher Scientific, Waltham, Massachusetts, USA). Stably transfected cells were maintained with hygromycin B (0.2 mg/mL, Roche, Basel, Switzerland) or geneticin (0.4 mg/mL, Sigma-Aldrich, Darmstadt, Germany). All cells were routinely tested using the Mycoplasma kit detection from Applied Biological Material (Richmond, Canada).

### P2X7 activity assays

Measurement of intracellular calcium concentration with FURA-2AM and of plasma membrane permeabilization to ethidium bromide was performed as previously described [82].

### Confocal microscopy

CT26 and HCT116 cells were seeded onto 24 mm glass coverslips (Thermo Fisher Scientific), and loaded with 1 µM plasma membrane dye PKH26GL (Sigma-Aldrich) or FM4-64 (Thermo Fischer Scientific) plus 1 µM Quinacrine dihydrochloride (Q3251, Sigma Aldrich) for ATP and nucleic acid staining. Live single-cell imaging was performed with an Olympus FV3000 confocal microscope using the FV31S-SW software (Olympus, Tokyo, Japan). The excitation wavelengths were 445 nm and 594 nm, respectively. Emission was measured in the 610–710 nm range for red staining and in the 460–500 nm range for green staining. Untreated CT26 and CT26 cells pre-treated for 10 min with the P2X7 antagonist AZ10606120 (5 µM) were stimulated with BzATP 300 µM. Images were acquired before (time 0) and following stimulation for a total of 30 min.

### Vesicles concentration

Vesicles were concentrated from the cell culture supernatant by centrifugation. 5 x 10^6^ cells were seeded in T175 flasks and after 24 h, the medium was removed, and cells were left adherent and washed with PBS. Then they were incubated in 10 ml of “saline solution” (125 mM NaCl, 5 mM KCl, 1 mM MgSO_4,_ 1 mM NaH_2_PO_4_, 20 mM HEPES, 5.5 mM glucose, 5 mM NaHCO_3_, 1 mM CaCl_2_, pH 7.4) with or without 3 mM ATP (pH 7.4) for 30 min at 37 °C with 5% CO_2,_ and in some conditions, pre-treated for 10 min with the P2X7 antagonists AZ10606120 (5 µM) (Tocris Bioscience, Bristol, UK) or A740003 (20 µM) (Tocris Bioscience). The supernatant was first depleted of cells and debris by centrifugation at 2000 x g for 10 min at 4 °C. The vesicles were then pelleted at 20000 x g for one hour at 4 °C. When the vesicles were collected to treat other cells, they underwent an extra wash at 20,000 x g in PBS for one hour at 4 °C to remove any possible remaining agonist or antagonist traces.

### ATP and adenosine measure

2 × 10^4^ CT26 pmeLUC cells were seeded in a 96-well plate and used as the ATP sensors. Spontaneously released (S-VS) or ATP-stimulated (P2X7-VS) vesicles were collected from CT26 cells and washed as described above, and 4 x 10^9^ vesicles were resuspended in 10 µL of PBS. D- luciferin (Promega, Madison, Wisconsin, USA) was added to the wells containing CT26 pmeLUC at a concentration of 60 µg/mL. Basal luminescence emission was measured for 5 min using an IVIS Lumina Luminometer (Perkin Elmer, Waltham, Massachusetts, USA). Vesicles, 10 µL of PBS or vehicle, were added to the cells, and luminescence emission was acquired for additional 5 min. Photon emission was quantified using the Living Image^®^ Software (Perkin Elmer) as total photons/seconds (p/s). Changes in ATP concentration were expressed as a fold increase on basal luminescence emission. The vehicle used to obtain data shown in Fig.1I, J was the “saline solution” containing originally 3 mM ATP that has been subjected to the same manipulations of the “saline solution” used to isolate vesicles (see above), including centrifugation to remove the supernatant, followed by a wash with fresh PBS not containing any ATP, this vehicle was therefore virtually ATP free. ATP and adenosine levels were measured in the supernatant of CT26 cells 30 and 90 min after the addition of vehicle, S-VS, or P2X7-VS or P2X7-VS plus AB-680 (5 µM, MCE Medchem Express, distributed by DBA Italia) using ENLITEN rLuciferin/Luciferase reagent (Promega) [7] or adenosine assay kit [83] (Cell Biolabs, Inc. MET-5090, San Diego, California, USA).

### Western blot

CT26 cells, HCT116 cells, S-VS and P2X7-VS were collected in RIPA buffer plus Halt™ Protease and Phosphatase inhibitor cocktail EDTA-free 100 x (Sigma-Aldrich). Lungs were homogenized in lysis buffer (300 µM sucrose, 1 mM K_2_HPO_4_, 5,5 mM glucose, 20 mM HEPES pH 7,4, Halt^TM^ Protease and Phosphatase inhibitor cocktail EDTA-free 100 x, IGEPAL CA-630 0.5%). Protein lysates were loaded and separated on 4-12% NuPAGE Bis-Tris precast gels (Thermo Fisher Scientific) and transferred onto a nitrocellulose membrane, and incubated overnight with the following primary antibodies: anti-GM130 1:500 (74220, Exosomal Marker Antibody Sample Kit, Cell Signaling Technology), anti-Alix 1:500 (74,220, Exosomal Marker Antibody Sample Kit, Cell Signaling Technology, Danvers, Massachusetts, USA), anti-P2X7 1:300 (P8232, Sigma Aldrich) anti-CD73 1:250 (Bs-4834R, Bioss Antibodies, Woburn, Massachusetts, USA), anti-CD39 1:500 (CQA2933, Cohesion Bioscience, London, UK) anti-A2A (SC32261, Santa Cruz Biotechnology, Dallas, Texas, USA), anti-FOXP3 1:500 (14-5773-82, Thermo Fisher Scientific) and anti-myosin 1:1000 (3403S, Cell Signaling Technology). Incubation with secondary anti-rabbit (A16096, Thermo Fisher Scientific) or anti-mouse (62-6520, Thermo Fisher Scientific) antibodies (1:2000) was performed for 1 h. Protein bands were visualized using the ECL HRP Chemiluminescent Substrate ETA *C* ULTRA 2.0 (Cyanagen Srl, Bologna, Italy) with a Licor C-Digit Model 3600.

### Nanoparticle tracking analysis

Vesicles were resuspended in 60 µL of filtered PBS and diluted 1:100 or 1:500 in PBS. Vesicles were tracked using the Nanosight system (Nanosight LM10, Malvern, UK), according to the manufacturer’s instructions. Data were analyzed using the NanoSight Software NTA 3.2 Dev Build 3.2.16. Vesicles present in mice’s plasma were quantified using Videodrop Technology (Myriade). The samples were diluted 1:100 in PBS.

### Scratch recovery assay

A Wound Healing Assay (ab242285, Abcam, Cambridge, UK) was used for scratch tests in serum-free RPMI 1640 medium. CT26 cells were seeded in 24-well plates containing inserts. When they reached confluence, the insert was removed to generate a 0.9 mm wound field. Cells were treated with 10 µl of PBS (control), S-VS, P2X7-VS, or AZ-VS. Pictures of scratches were acquired at time 0 (T0) and after 24 and 48 h (T24, T48) with a phase-contrast optical DM IL Led Leica Microsystem microscope (LEICA ICC50 HD, Wetzlar, Germany). ImageJ Software was used for scratch measurement. The percentage closure was calculated by comparison with T0.

### Murine models

A syngeneic experimental metastasis model was established by injecting 2.5 x 10^5^ CT26 Luc2 cells into the tail vein of BALB/c 4-6 weeks-old female mice (Envigo, Udine, Italy). In the first set of experiments, CT26 Luc2 cells were detached from the flasks, counted, and incubated with 4 x 10^9^ vesicles collected from another batch of the same cells as described in the “Vesicles concentration” paragraph and following literature guidelines [84]. The cell plus vesicle suspension was intravenously injected into the mice. Cell dissemination was monitored every 72 h by measuring luciferase Luc2 photon emission using an IVIS Lumina Luminometer (Perkin Elmer) as described previously [6]. In a second set of experiments, animals were treated every 72 h with an i.p. injection of placebo (PBS + 0.002% DMSO), the P2X7 antagonist AZ10606120 (2 mg/Kg) (Tocris Bioscience), the A2A antagonist SCH58261 (1 mg/Kg) (Tocris Bioscience), and a combination of both antagonists. The same antagonist administration schedule was also employed in non tumor-bearing BALB/c mice and in a xenotransplant model obtained by tail vein injection of 2 x 10^6^ HCT116-Luc2 cells in *nude/nude* mice (Envigo). A subcutaneous tumor model was obtained by injecting 5 x 10^5^ CT26 cells into the flank of 6-week-old BALB/c female mice (Envigo). After five days, when tumor masses became evident, we started the treatment, which was repeated every 72 h, with an intraperitoneal (i.p.) injection of placebo (PBS + 0.002% DMSO), the P2X7 antagonist AZ10606120 (2 mg/Kg) (Tocris Bioscience), the A2A antagonist SCH58261 (1 mg/Kg) (Tocris Bioscience), and a combination of both antagonists. Tumor size was determined as described in [7]. The same antagonist administration schedule was also employed in a xenotransplant model obtained by subcutaneous injection of 2 x 10^6^ HCT116-Luc2 cells in *nude/nude* mice (Envigo). For all experiments, mice were randomized into four groups, and the operator was blinded to the allocation group. Blood samples (250 µl) were collected from the submandibular vein and supplemented with 1.5 mg/ml EDTA, immediately before sacrifice. Plasma was separated by centrifugation (1000 x g, 5 min at 4 °C), supplemented with Halt Protease and Phosphatase Inhibitor Cocktail (Thermo Scientific), and stored at – 80 °C [7]. All animal procedures were approved by the University of Ferrara Ethics Committee and the Italian Ministry of Health (Italian D. Lgs 26/204) and were in accordance with generally accepted guidelines for the welfare and use of animals in cancer research [85]. Tissue slides from the colons of WT and PIRC rats and related tumors were obtained from archived materials available at Caderni’s laboratory [40, 41].

### Cytokine quantification

Plasma samples were diluted 1:2 with the sample buffer provided by the ELISA kit, and levels of interleukin 17 were measured using the Simple Plex™ Cartridge Kit with the Ella Automated Immunoassay System (Biotechne, Minneapolis, Minnesota, USA). Interleukin 23 was quantified using the mouse IL-23 Quantikine ELISA kit according to the manufacturer’s instructions (R&D Systems).

### Histology

Tissue slides from mouse lungs were processed as previously described [4]. Images were acquired using the NIS-Element Software with a Nikon Eclipse H550L microscope (Nikon Europe, Amstelveen, Netherlands). Metastasis formation in the lungs was analyzed by quantifying the area of the samples covered by tumor cells, creating a compact tumor mass area as opposed to the typical alveolar structure of the lungs by hematoxylin/eosin staining. The total area of metastasis is expressed as a percentage of the entire lung area. The areas were quantified using ImageJ Software.

### Immunohistochemistry

Tissue slides from the mouse lungs and rat colons were analyzed for P2X7, CD39, CD73, and A2A expression using the following primary antibodies: P2X7 1:100 (P8232, Sigma-Aldrich), CD39 1:100 (NBP2-67230, Novus Biologicals, Minneapolis, Minnesota, USA), CD73 1:500 (MAB5795, R&D Systems, Minneapolis, Minnesota, USA), and A2A 1:100 (SC32261, Santa Cruz Biotechnology). The protocol used for tissue staining has been described previously [14]. Images were acquired using a Nikon Eclipse H550L microscope and the NIS-Element Software (Nikon). The percentage of positive cells was quantified using QuPath open-source software for bioimage analysis. Cells were identified with the cell detection function, setting minimum and maximum area at 200 and 2500 pixels, respectively. Subsequently, DAB-positive cells were evaluated with the set cell intensity function fixing a 0,25 threshold for DAB, as suggested by the developers [86].

### Quantitative real-time PCR

Samples from human colon carcinoma patients were obtained from five commercial arrays (TissueScan™ cDNA arrays HCRT301, HCRT302, HCRT303, HCRT304, HCRT305, OriGene, Rockville, Maryland, USA). Samples in which mRNA levels for housekeeping genes were not detectable were excluded from the analysis. A total of 158 patients were analyzed and subdivided according to the diagnostic phase into stage I (*n* = 24), stage II (*n* = 50), stage III (*n* = 52) and stage IV (*n* = 32). Stage IV patient data were further analyzed according to the origin of the cDNA from the primary (*n* = 21) or metastatic (*n* = 11) specimens. CRC samples cDNA were used as a template for quantitative Real-Time PCR (qRT-PCR) using TaqMan®MGB probes, FAM™ dye-labeled (20X), and TaqMan Gene Expression Master Mix 2X (Applied Biosystems, Waltham, Massachusetts, USA). Taqman custom probes and primers for P2X7A and P2X7B were previously described by Pegoraro et al. [78]. Taqman predesigned probes for the other genes were, respectively: Hs00969556_m1 ENTPD1 for CD39, Hs00159686_m1 NT5E for CD73, and Hs00169123_m1 ADORA2A for A2A (Applied Biosystems). Glyceraldehyde-3-phosphate dehydrogenase (Hs99999905_m1, Applied Biosystems) was used as a housekeeping gene. qRT-PCR was performed using an AB PRISM 7300 Step One Real-Time PCR system (Applied Biosystems) as previously described [6]. Formalin-fixed paraffin-embedded tumor samples (*n* = 12) from patients with colon carcinoma were collected according to a protocol approved by the Istituto Romagnolo per lo studio dei tumori (IRST) Ethics Committee (CEROM IRST IRCCS-AVR, protocol code: IRST B125 approved on the 19^th^ of March 2021). All patients signed an informed consent form before surgery. No specific inclusion or exclusion criteria were set. Patients’ information is summarized in Supplementary Table 1. Patients were stratified according to *APC* mutational status evaluated by whole exome sequencing (*APC* variants were considered with a variant allele frequency of 5% or greater). Total RNA was extracted from paraffin-embedded tissue sections using the Maxwell RSC RNA FFPE Kit (AS1440, Promega) on a Maxwell CSC 48 automated nucleic acid extraction system (Promega) according to manufacturer instructions. Total RNA was quantified using the Qubit RNA High Sensitivity (HS) Assay Kit (Q32852, Invitrogen). 200 ng of total RNA were reverse-transcribed using the SuperScript VILO cDNA Synthesis Kit (Q32852, Invitrogen). Real-time PCR was performed using the 7500 Real-time PCR System (Applied Biosystem, USA) as previously described [79].

### Gene expression correlation

Spearman’s correlation analysis among the genes tested in our stage IV metastatic patients’ was performed using GraphPad Prism Software (GraphPad, La Jolla, California, USA). Moreover, P2X7 and A2A gene expression was evaluated in colon adenocarcinoma samples from the Cancer Genome Atlas database (TGCA) using the GEPIA web server [87]. Spearman’s correlation coefficient was applied for the analysis calculation with a non-log scale, whereas the data were visualized using the log-scale axis.

### Statistics

All data are shown as mean ± standard error of the mean (SEM). Significance was calculated assuming equal standard deviation and variance, with a two-tailed Student’s t-test, ordinary one- or two way ANOVA performed using GraphPad Prism Software (GraphPad). For each in vivo experiment, the group size and statistical power were selected following computation a priori, based on previous data [6, 7, 78], using an online sample size calculator (clincalc.com/stats/simplesize.aspx). Statistical significance was set at P-values lower than 0.05.

## Supplementary information

Supplementary Figure 1

Supplementary Figure 2

Supplementary Figure 3

Supplemental material legends

movie 1

movie 2

movie 3

Supplementary Table I

## Data Availability

The data sets used and/or analysed during the current study are available from the corresponding author on reasonable request.

## References

[1] Di Virgilio F, Sarti AC, Falzoni S, De Marchi E, Adinolfi E. Extracellular ATP and P2 purinergic signalling in the tumour microenvironment. Nat Rev Cancer. 2018;18:601–18. 30006588 10.1038/s41568-018-0037-0

[2] Chiarella AM, Ryu YK, Manji GA, Rustgi AK. Extracellular ATP and adenosine in cancer pathogenesis and treatment. Trends Cancer. 2021;7:731–50. 34074623 10.1016/j.trecan.2021.04.008

[3] Bai X, Li Q, Peng X, Li X, Qiao C, Tang Y, et al. P2X7 receptor promotes migration and invasion of non-small cell lung cancer A549 cells through the PI3K/Akt pathways. Purinergic Signal. 2023;19:685–97. 10.1007/s11302-023-09928-z PMC10754800 36854856

[4] Adinolfi E, Raffaghello L, Giuliani AL, Cavazzini L, Capece M, Chiozzi P, et al. Expression of P2X7 receptor increases in vivo tumor growth. Cancer Res. 2012;72:2957–69. 22505653 10.1158/0008-5472.CAN-11-1947

[5] Amoroso F, Capece M, Rotondo A, Cangelosi D, Ferracin M, Franceschini A, et al. The P2X7 receptor is a key modulator of the PI3K/GSK3beta/VEGF signaling network: evidence in experimental neuroblastoma. Oncogene. 2015;34:5240–51. 25619831 10.1038/onc.2014.444

[6] Pegoraro A, De Marchi E, Ferracin M, Orioli E, Zanoni M, Bassi C, et al. P2X7 promotes metastatic spreading and triggers release of miRNA-containing exosomes and microvesicles from melanoma cells. Cell Death Dis. 2021;12:1088. 34789738 10.1038/s41419-021-04378-0 PMC8599616

[7] De Marchi E, Orioli E, Pegoraro A, Sangaletti S, Portararo P, Curti A, et al. The P2X7 receptor modulates immune cells infiltration, ectonucleotidases expression and extracellular ATP levels in the tumor microenvironment. Oncogene. 2019;38:3636–50. 30655604 10.1038/s41388-019-0684-y PMC6756114

[8] Adinolfi E, De Marchi E, Orioli E, Pegoraro A, Di Virgilio F. Role of the P2X7 receptor in tumor-associated inflammation. Curr Opin Pharm. 2019;47:59–64. 10.1016/j.coph.2019.02.012 30921559

[9] Kepp O, Bezu L, Yamazaki T, Di Virgilio F, Smyth MJ, Kroemer G, et al. ATP and cancer immunosurveillance. EMBO J. 2021;40:e108130. 34121201 10.15252/embj.2021108130 PMC8246257

[10] Yegutkin GG, Boison D. ATP and adenosine metabolism in cancer: exploitation for therapeutic gain. Pharmacol Rev. 2022;74:797–822. 35738682 10.1124/pharmrev.121.000528 PMC9553103

[11] Zanoni M, Pegoraro A, Adinolfi E, De Marchi E. Emerging roles of purinergic signaling in anti-cancer therapy resistance. Front Cell Dev Biol. 2022;10:1006384. 36200041 10.3389/fcell.2022.1006384 PMC9527280

[12] Merighi S, Battistello E, Giacomelli L, Varani K, Vincenzi F, Borea PA, et al. Targeting A3 and A2A adenosine receptors in the fight against cancer. Expert Opin Ther Targets. 2019;23:669–78. 31189400 10.1080/14728222.2019.1630380

[13] de Araujo JB, Kerkhoff VV, de Oliveira Maciel SFV, de Resende ESDT. Targeting the purinergic pathway in breast cancer and its therapeutic applications. Purinergic Signal. 2021;17:179–200. 33576905 10.1007/s11302-020-09760-9 PMC7879595

[14] De Marchi E, Pegoraro A, Turiello R, Di Virgilio F, Morello S, Adinolfi E. A2A Receptor contributes to tumor progression in P2X7 null mice. Front Cell Dev Biol. 2022;10:876510. 35663396 10.3389/fcell.2022.876510 PMC9159855

[15] Yan J, Li XY, Roman Aguilera A, Xiao C, Jacoberger-Foissac C, Nowlan B, et al. Control of metastases via Myeloid CD39 and NK Cell effector function. Cancer Immunol Res. 2020;8:356–67. 31992567 10.1158/2326-6066.CIR-19-0749

[16] Casey M, Segawa K, Law SC, Sabdia MB, Nowlan B, Salik B, et al. Inhibition of CD39 unleashes macrophage antibody-dependent cellular phagocytosis against B-cell lymphoma. Leukemia. 2023;37:379–87. 36539557 10.1038/s41375-022-01794-9

[17] Li XY, Moesta AK, Xiao C, Nakamura K, Casey M, Zhang H, et al. Targeting CD39 in cancer reveals an extracellular ATP- and inflammasome-driven tumor immunity. Cancer Discov. 2019;9:1754–73. 31699796 10.1158/2159-8290.CD-19-0541 PMC6891207

[18] Demeules M, Scarpitta A, Hardet R, Gonde H, Abad C, Blandin M, et al. Evaluation of nanobody-based biologics targeting purinergic checkpoints in tumor models in vivo. Front Immunol. 2022;13:1012534. 36341324 10.3389/fimmu.2022.1012534 PMC9626963

[19] Sohal IS, Kasinski AL. Emerging diversity in extracellular vesicles and their roles in cancer. Front Oncol. 2023;13:1167717. 37397375 10.3389/fonc.2023.1167717 PMC10312242

[20] Jeppesen DK, Zhang Q, Franklin JL, Coffey RJ. Extracellular vesicles and nanoparticles: emerging complexities. Trends Cell Biol. 2023;33:667–81. 36737375 10.1016/j.tcb.2023.01.002 PMC10363204

[21] Becker A, Thakur BK, Weiss JM, Kim HS, Peinado H, Lyden D. Extracellular Vesicles in Cancer: Cell-to-Cell Mediators of Metastasis. Cancer Cell. 2016;30:836–48. 27960084 10.1016/j.ccell.2016.10.009 PMC5157696

[22] Catalano M, O’Driscoll L. Inhibiting extracellular vesicles formation and release: a review of EV inhibitors. J Extracell Vesicles. 2020;9:1703244. 32002167 10.1080/20013078.2019.1703244 PMC6968539

[23] Lombardi M, Gabrielli M, Adinolfi E, Verderio C. Role of ATP in Extracellular vesicle biogenesis and dynamics. Front Pharm. 2021;12:654023. 10.3389/fphar.2021.654023 PMC8006391 33790800

[24] D’Arrigo G, Gabrielli M, Scaroni F, Swuec P, Amin L, Pegoraro A, et al. Astrocytes-derived extracellular vesicles in motion at the neuron surface: Involvement of the prion protein. J Extracell Vesicles. 2021;10:e12114. 34276899 10.1002/jev2.12114 PMC8275823

[25] Vultaggio-Poma V, Falzoni S, Chiozzi P, Sarti AC, Adinolfi E, Giuliani AL, et al. Extracellular ATP is increased by release of ATP-loaded microparticles triggered by nutrient deprivation. Theranostics. 2022;12:859–74. 34976217 10.7150/thno.66274 PMC8692914

[26] Carotti V, Rigalli JP, van Asbeck-van der Wijst J, Hoenderop JGJ. Interplay between purinergic signalling and extracellular vesicles in health and disease. Biochem Pharm. 2022;203:115192. 35905971 10.1016/j.bcp.2022.115192

[27] Sedlak JC, Yilmaz OH, Roper J. Metabolism and Colorectal Cancer. Annu Rev Pathol. 2023;18:467–92. 36323004 10.1146/annurev-pathmechdis-031521-041113 PMC9877174

[28] Waldum H, Fossmark R. Inflammation and digestive cancer. Int J Mol Sci.2023;24:13503. 37686307 10.3390/ijms241713503 PMC10487643

[29] Shin AE, Giancotti FG, Rustgi AK. Metastatic colorectal cancer: mechanisms and emerging therapeutics. Trends Pharm Sci. 2023;44:222–36. 36828759 10.1016/j.tips.2023.01.003 PMC10365888

[30] Cancer Genome Atlas N. Comprehensive molecular characterization of human colon and rectal cancer. Nature. 2012;487:330–7. 22810696 10.1038/nature11252 PMC3401966

[31] Kunzli BM, Bernlochner MI, Rath S, Kaser S, Csizmadia E, Enjyoji K, et al. Impact of CD39 and purinergic signalling on the growth and metastasis of colorectal cancer. Purinergic Signal. 2011;7:231–41. 21484085 10.1007/s11302-011-9228-9 PMC3146639

[32] Calik I, Calik M, Turken G, Ozercan IH. A promising independent prognostic biomarker in colorectal cancer: P2X7 receptor. Int J Clin Exp Pathol. 2020;13:107–21. 32211091 PMC7061807

[33] Feng Y, Xu X, Zhang J, Sanderson C, Xia J, Bu Z, et al. CD39(+) tumor infiltrating T cells from colorectal cancers exhibit dysfunctional phenotype. Am J Cancer Res. 2024;14:585–600. 38455401 10.62347/IZEN3736 PMC10915329

[34] Messaoudi N, Cousineau I, Arslanian E, Henault D, Stephen D, Vandenbroucke-Menu F, et al. Prognostic value of CD73 expression in resected colorectal cancer liver metastasis. Oncoimmunology. 2020;9:1746138. 32363113 10.1080/2162402X.2020.1746138 PMC7185220

[35] Ye H, Zhao J, Xu X, Zhang D, Shen H, Wang S. Role of adenosine A2a receptor in cancers and autoimmune diseases. Immun Inflamm Dis. 2023;11:e826. 37102661 10.1002/iid3.826 PMC10091380

[36] De Marchi E, Orioli E, Pegoraro A, Adinolfi E, Di Virgilio F. Detection of Extracellular ATP in the Tumor Microenvironment, Using the pmeLUC Biosensor. Methods Mol Biol. 2020;2041:183–95. 31646489 10.1007/978-1-4939-9717-6_13

[37] Downs-Canner S, Berkey S, Delgoffe GM, Edwards RP, Curiel T, Odunsi K, et al. Suppressive IL-17A(+)Foxp3(+) and ex-Th17 IL-17A(neg)Foxp3(+) T(reg) cells are a source of tumour-associated T(reg) cells. Nat Commun. 2017;8:14649. 28290453 10.1038/ncomms14649 PMC5355894

[38] Pegoraro A, De Marchi E, Adinolfi E. P2X7 Variants in oncogenesis. Cells. 2021;10:189. 33477845 10.3390/cells10010189 PMC7832898

[39] Adinolfi E, De Marchi E, Grignolo M, Szymczak B, Pegoraro A. The P2X7 Receptor in oncogenesis and metastatic dissemination: new insights on vesicular release and adenosinergic crosstalk. Int J Mol Sci. 2023;24:13906. 10.3390/ijms241813906 PMC10531279 37762206

[40] Femia AP, Soares PV, Luceri C, Lodovici M, Giannini A, Caderni G. Sulindac, 3,3’-diindolylmethane and curcumin reduce carcinogenesis in the Pirc rat, an Apc-driven model of colon carcinogenesis. BMC Cancer. 2015;15:611. 26335331 10.1186/s12885-015-1627-9 PMC4559292

[41] Vitali F, Tortora K, Di Paola M, Bartolucci G, Menicatti M, De Filippo C, et al. Intestinal microbiota profiles in a genetic model of colon tumorigenesis correlates with colon cancer biomarkers. Sci Rep. 2022;12:1432. 35082322 10.1038/s41598-022-05249-0 PMC8792020

[42] Cervantes A, Adam R, Rosello S, Arnold D, Normanno N, Taieb J, et al. Metastatic colorectal cancer: ESMO Clinical Practice Guideline for diagnosis, treatment and follow-up. Ann Oncol. 2023;34:10–32. 36307056 10.1016/j.annonc.2022.10.003

[43] Zeineddine FA, Zeineddine MA, Yousef A, Gu Y, Chowdhury S, Dasari A, et al. Survival improvement for patients with metastatic colorectal cancer over twenty years. NPJ Precis Oncol. 2023;7:16. 36781990 10.1038/s41698-023-00353-4 PMC9925745

[44] Bekaii-Saab TS, Barzi A, Cusnir M. Improving survival in metastatic colorectal cancer through optimized patient selection. Clin Adv Hematol Oncol. 2024;22:1–20. 38805297

[45] Urabe F, Patil K, Ramm GA, Ochiya T, Soekmadji C. Extracellular vesicles in the development of organ-specific metastasis. J Extracell Vesicles. 2021;10:e12125. 34295457 10.1002/jev2.12125 PMC8287318

[46] D’Antongiovanni V, Fornai M, Pellegrini C, Benvenuti L, Blandizzi C, Antonioli L. The adenosine system at the crossroads of intestinal inflammation and neoplasia. Int J Mol Sci. 2020;21:5089. 10.3390/ijms21145089 PMC7403993 32708507

[47] Welsh JA, Goberdhan DCI, O’Driscoll L, Buzas EI, Blenkiron C, Bussolati B, et al. Minimal information for studies of extracellular vesicles (MISEV2023): From basic to advanced approaches. J Extracell Vesicles. 2024;13:e12404. 38326288 10.1002/jev2.12404 PMC10850029

[48] Maacha S, Bhat AA, Jimenez L, Raza A, Haris M, Uddin S, et al. Extracellular vesicles-mediated intercellular communication: roles in the tumor microenvironment and anti-cancer drug resistance. Mol Cancer. 2019;18:55. 30925923 10.1186/s12943-019-0965-7 PMC6441157

[49] Irep N, Inci K, Tokgun PE, Tokgun O. Exosome inhibition improves response to first-line therapy in small cell lung cancer. J Cell Mol Med. 2024;28:e18138. 38353469 10.1111/jcmm.18138 PMC10865916

[50] Falzoni S, Vultaggio-Poma V, Chiozzi P, Tarantini M, Adinolfi E, Boldrini P, et al. The P2X7 Receptor is a master regulator of microparticle and mitochondria exchange in mouse microglia. Function. 2024;5.4:zqae019. 10.1093/function/zqae019 PMC11237899 38984997

[51] Pizzirani C, Ferrari D, Chiozzi P, Adinolfi E, Sandona D, Savaglio E, et al. Stimulation of P2 receptors causes release of IL-1beta-loaded microvesicles from human dendritic cells. Blood. 2007;109:3856–64. 17192399 10.1182/blood-2005-06-031377

[52] Longo Y, Mascaraque SM, Andreacchio G, Werner J, Katahira I, De Marchi E, et al. The purinergic receptor P2X7 as a modulator of viral vector-mediated antigen cross-presentation. Front Immunol. 2024;15:1360140. 38711513 10.3389/fimmu.2024.1360140 PMC11070468

[53] Iqbal J, Bano S, Khan IA, Huang Q. A patent review of P2X7 receptor antagonists to treat inflammatory diseases (2018-present). Expert Opin Ther Pat. 2024;34:263–71. 38828613 10.1080/13543776.2024.2363885

[54] Huangfu L, Li R, Huang Y, Wang S. The IL-17 family in diseases: from bench to bedside. Signal Transduct Target Ther. 2023;8:402. 37816755 10.1038/s41392-023-01620-3 PMC10564932

[55] Razi S, Baradaran Noveiry B, Keshavarz-Fathi M, Rezaei N. IL-17 and colorectal cancer: From carcinogenesis to treatment. Cytokine. 2019;116:7–12. 30684916 10.1016/j.cyto.2018.12.021

[56] Ghoreschi K, Balato A, Enerback C, Sabat R. Therapeutics targeting the IL-23 and IL-17 pathway in psoriasis. Lancet. 2021;397:754–66. 33515492 10.1016/S0140-6736(21)00184-7

[57] Liu C, Liu R, Wang B, Lian J, Yao Y, Sun H, et al. Blocking IL-17A enhances tumor response to anti-PD-1 immunotherapy in microsatellite stable colorectal cancer. J Immunother Cancer. 2021;9:e001895. 10.1136/jitc-2020-001895 PMC7813395 33462141

[58] Wight AE, Sido JM, Degryse S, Ao L, Nakagawa H, Qiu Vivian Y, et al. Antibody-mediated blockade of the IL23 receptor destabilizes intratumoral regulatory T cells and enhances immunotherapy. Proc Natl Acad Sci USA. 2022;119:e2200757119. 35482921 10.1073/pnas.2200757119 PMC9170135

[59] Sharp SP, Avram D, Stain SC, Lee EC. Local and systemic Th17 immune response associated with advanced stage colon cancer. J Surg Res. 2017;208:180–6. 27993206 10.1016/j.jss.2016.09.038 PMC6086724

[60] D’Addio F, Vergani A, Potena L, Maestroni A, Usuelli V, Ben Nasr M, et al. P2X7R mutation disrupts the NLRP3-mediated Th program and predicts poor cardiac allograft outcomes. J Clin Invest. 2018;128:3490–503. 30010623 10.1172/JCI94524 PMC6063506

[61] Tokano M, Matsushita S, Takagi R, Yamamoto T, Kawano M. Extracellular adenosine induces hypersecretion of IL-17A by T-helper 17 cells through the adenosine A2a receptor. Brain Behav Immun Health. 2022;26:100544. 36467126 10.1016/j.bbih.2022.100544 PMC9712818

[62] Wang L, Wan H, Tang W, Ni Y, Hou X, Pan L, et al. Critical roles of adenosine A2A receptor in regulating the balance of Treg/Th17 cells in allergic asthma. Clin Respir J. 2018;12:149–57. 27216911 10.1111/crj.12503

[63] Diaz-Perez JA, Killeen ME, Yang Y, Carey CD, Falo LD Jr., Mathers AR. Extracellular ATP and IL-23 Form a Local Inflammatory Circuit Leading to the Development of a Neutrophil-Dependent Psoriasiform Dermatitis. J Invest Dermatol. 2018;138:2595–605. 29870687 10.1016/j.jid.2018.05.018 PMC6251745

[64] Akhtari M, Vojdanian M, Javinani A, Ashraf-Ganjouei A, Jamshidi A, Mahmoudi M. Activation of adenosine A(2A) receptor induced interleukin-23 mRNA expression in macrophages of ankylosing spondylitis patients. Cytokine. 2020;128:154997. 31978612 10.1016/j.cyto.2020.154997

[65] Delgado-Ramirez Y, Baltazar-Perez I, Martinez Y, Callejas BE, Medina-Andrade I, Olguin JE, et al. STAT1 Is Required for decreasing accumulation of granulocytic cells via IL-17 during initial steps of colitis-associated cancer. Int J Mol Sci. 2021;22.14:7695 10.3390/ijms22147695 PMC8306338 34299314

[66] Qi H, Yang H, Xu G, Ren J, Hua W, Shi Y, et al. Therapeutic efficacy of IL-17A antibody injection in preventing the development of colitis associated carcinogenesis in mice. Immunobiology. 2015;220:54–9. 25239511 10.1016/j.imbio.2014.09.002

[67] Chung AS, Wu X, Zhuang G, Ngu H, Kasman I, Zhang J, et al. An interleukin-17-mediated paracrine network promotes tumor resistance to anti-angiogenic therapy. Nat Med. 2013;19:1114–23. 23913124 10.1038/nm.3291

[68] Sinicrope FA, Rego RL, Ansell SM, Knutson KL, Foster NR, Sargent DJ. Intraepithelial effector (CD3+)/regulatory (FoxP3+) T-cell ratio predicts a clinical outcome of human colon carcinoma. Gastroenterology. 2009;137:1270–9. 19577568 10.1053/j.gastro.2009.06.053 PMC2873775

[69] Wang Z, Zhang J. FOXP3 promotes colorectal carcinoma liver metastases by evaluating MMP9 expression via regulating S-adenosylmethionine metabolism. Ann Transl Med. 2020;8:592. 32566619 10.21037/atm-20-3287 PMC7290543

[70] Di Virgilio F, Dal Ben D, Sarti AC, Giuliani AL, Falzoni S. The P2X7 Receptor in Infection and Inflammation. Immunity. 2017;47:15–31. 28723547 10.1016/j.immuni.2017.06.020

[71] Accogli T, Hibos C, Milian L, Geindreau M, Richard C, Humblin E, et al. The intrinsic expression of NLRP3 in Th17 cells promotes their protumor activity and conversion into Tregs. Cell Mol Immunol. 2025;22:541–56. 40195474 10.1038/s41423-025-01281-y PMC12041534

[72] Adinolfi E, Cirillo M, Woltersdorf R, Falzoni S, Chiozzi P, Pellegatti P, et al. Trophic activity of a naturally occurring truncated isoform of the P2X7 receptor. FASEB J. 2010;24:3393–404. 20453110 10.1096/fj.09-153601

[73] Pegoraro A, Grignolo M, Ruo L, Ricci L, Adinolfi E P2X7 variants in pathophysiology. Int J Mol Sci. 2024;25.12:6673 10.3390/ijms25126673 PMC11204217 38928378

[74] Ulrich H, Ratajczak MZ, Schneider G, Adinolfi E, Orioli E, Ferrazoli EG, et al. Kinin and purine signaling contributes to neuroblastoma metastasis. Front Pharm. 2018;9:500. 10.3389/fphar.2018.00500 PMC5968427 29867502

[75] Arnaud-Sampaio VF, Bento CA, Glaser T, Adinolfi E, Ulrich H, Lameu C. P2X7 receptor isoform B is a key drug resistance mediator for neuroblastoma. Front Oncol. 2022;12:966404. 36091161 10.3389/fonc.2022.966404 PMC9458077

[76] Tattersall L, Shah KM, Lath DL, Singh A, Down JM, De Marchi E, et al. The P2RX7B splice variant modulates osteosarcoma cell behaviour and metastatic properties. J Bone Oncol. 2021;31:100398. 35340569 10.1016/j.jbo.2021.100398 PMC8948168

[77] Giuliani AL, Colognesi D, Ricco T, Roncato C, Capece M, Amoroso F, et al. Trophic activity of human P2X7 receptor isoforms A and B in osteosarcoma. PLoS ONE. 2014;9:e107224. 25226385 10.1371/journal.pone.0107224 PMC4165768

[78] Pegoraro A, Orioli E, De Marchi E, Salvestrini V, Milani A, Di Virgilio F, et al. Differential sensitivity of acute myeloid leukemia cells to daunorubicin depends on P2X7A versus P2X7B receptor expression. Cell Death Dis. 2020;11:876. 33071281 10.1038/s41419-020-03058-9 PMC7569086

[79] Zanoni M, Sarti AC, Zamagni A, Cortesi M, Pignatta S, Arienti C, et al. Irradiation causes senescence, ATP release, and P2X7 receptor isoform switch in glioblastoma. Cell Death Dis. 2022;13:80. 35075119 10.1038/s41419-022-04526-0 PMC8786947

[80] Song H, Arredondo Carrera HM, Sprules A, Ji Y, Zhang T, He J, et al. C-terminal variants of the P2X7 receptor are associated with prostate cancer progression and bone metastasis - evidence from clinical and pre-clinical data. Cancer Commun. 2023;43:400–4. 10.1002/cac2.12391 PMC10009667 36582013

[81] Li B, Zhang G, Xu X. APC mutation correlated with poor response of immunotherapy in colon cancer. BMC Gastroenterol. 2023;23:95. 36977982 10.1186/s12876-023-02725-3 PMC10053134

[82] Di Virgilio F, Jiang LH, Roger S, Falzoni S, Sarti AC, Vultaggio-Poma V, et al. Structure, function and techniques of investigation of the P2X7 receptor (P2X7R) in mammalian cells. Methods Enzymol. 2019;629:115–50. 31727237 10.1016/bs.mie.2019.07.043

[83] Ocadlikova D, Fiordi B, Trabanelli S, Salvestrini V, Ciciarello M, Forte D,. et al. Non-canonical NF-kappaB signaling in dendritic cells is required for ATP-driven indoleamine 2,3-dioxygenase 1 induction through P2Y11 receptor. J Leukoc Biol. 2025;117:qiaF010. 39899472 10.1093/jleuko/qiaf010

[84] Kang M, Jordan V, Blenkiron C, Chamley LW. Biodistribution of extracellular vesicles following administration into animals: A systematic review. J Extracell Vesicles. 2021;10:e12085. 34194679 10.1002/jev2.12085 PMC8224174

[85] Workman P, Aboagye EO, Balkwill F, Balmain A, Bruder G, Chaplin DJ, et al. Guidelines for the welfare and use of animals in cancer research. Br J Cancer. 2010;102:1555–77. 20502460 10.1038/sj.bjc.6605642 PMC2883160

[86] Bankhead P, Loughrey MB, Fernandez JA, Dombrowski Y, McArt DG, Dunne PD, et al. QuPath: Open source software for digital pathology image analysis. Sci Rep. 2017;7:16878. 29203879 10.1038/s41598-017-17204-5 PMC5715110

[87] Tang Z, Li C, Kang B, Gao G, Li C, Zhang Z. GEPIA: a web server for cancer and normal gene expression profiling and interactive analyses. Nucleic Acids Res. 2017;45:W98–W102. 28407145 10.1093/nar/gkx247 PMC5570223

