# Supplementary figures and images for: P2X7 a new therapeutic target to block vesicle-dependent metastasis in colon carcinoma: Role of the A2A/CD39/CD73 axis
Source: Cell Death Dis. 2025 Aug 4;16(1):587. doi: 10.1038/s41419-025-07897-2 (PMC12322077; doi:10.1038/s41419-025-07897-2)

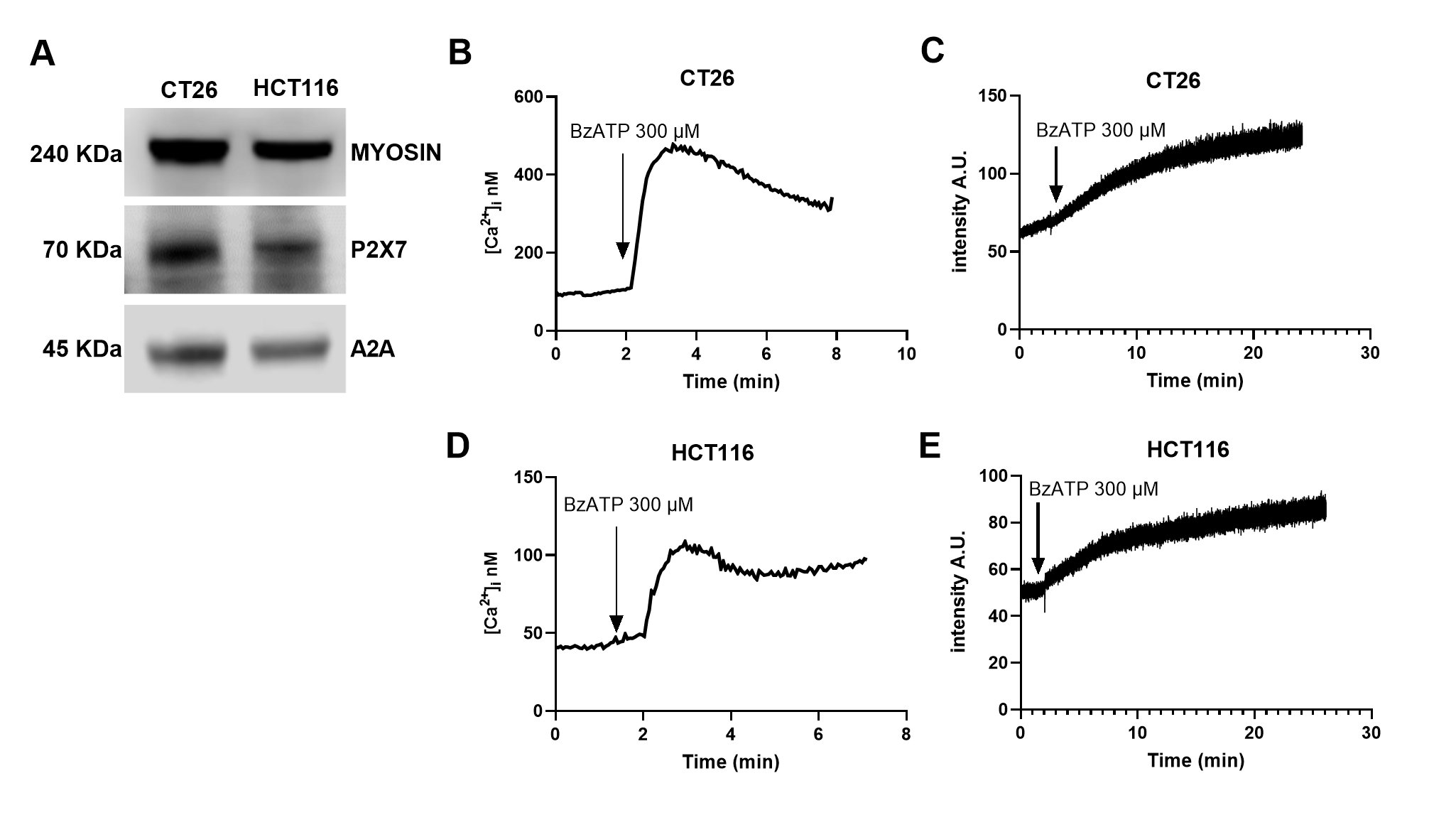

Supplement: Supplementary file 1 — Supplementary Figure 1 [file 41419_2025_7897_MOESM1_ESM.tif]

CD39

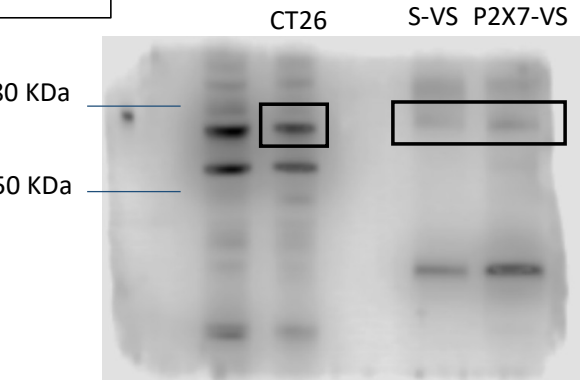

GM130

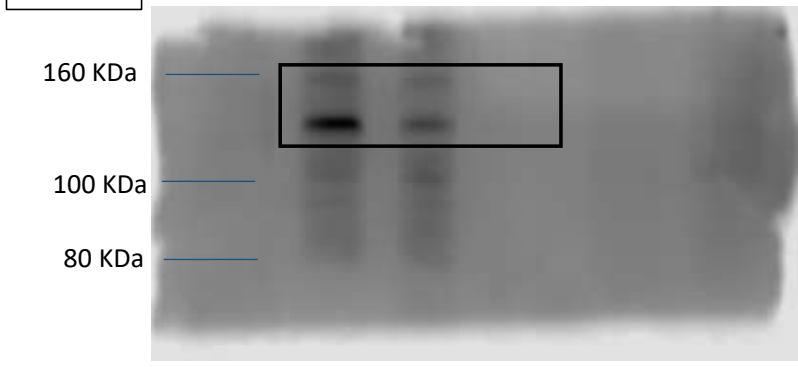

CD73

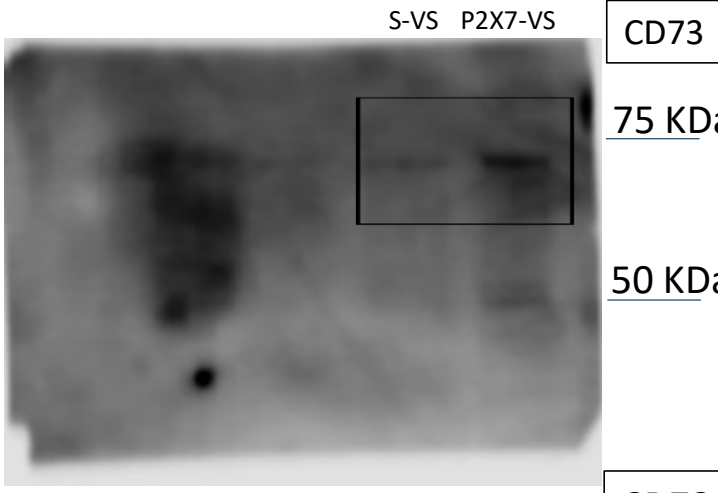

P2X7

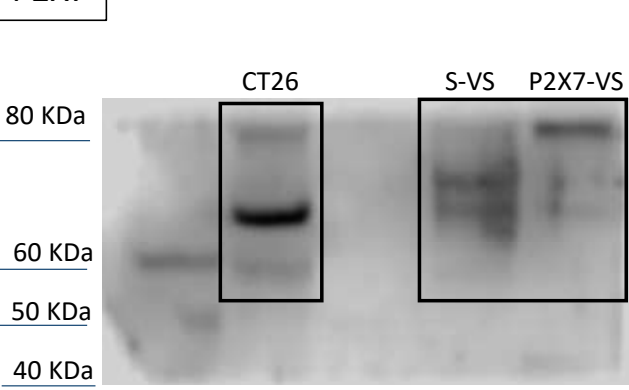

A2A

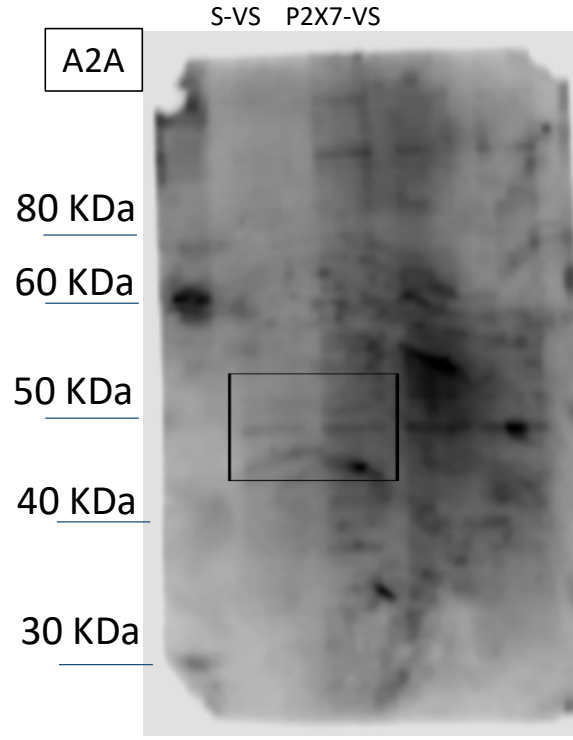

CD73

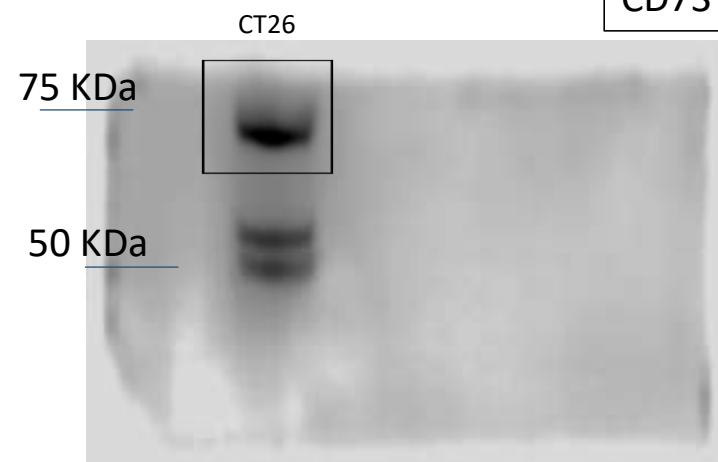

ALIX 1

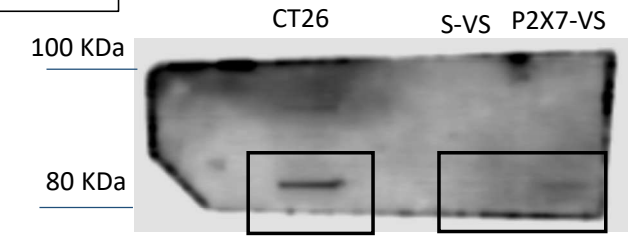

A2A

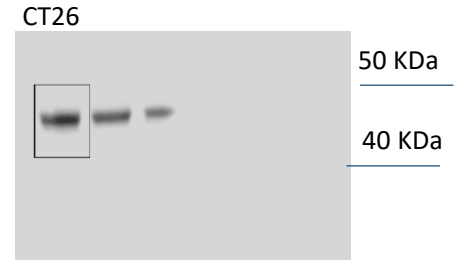

WB LUNGS figure 2

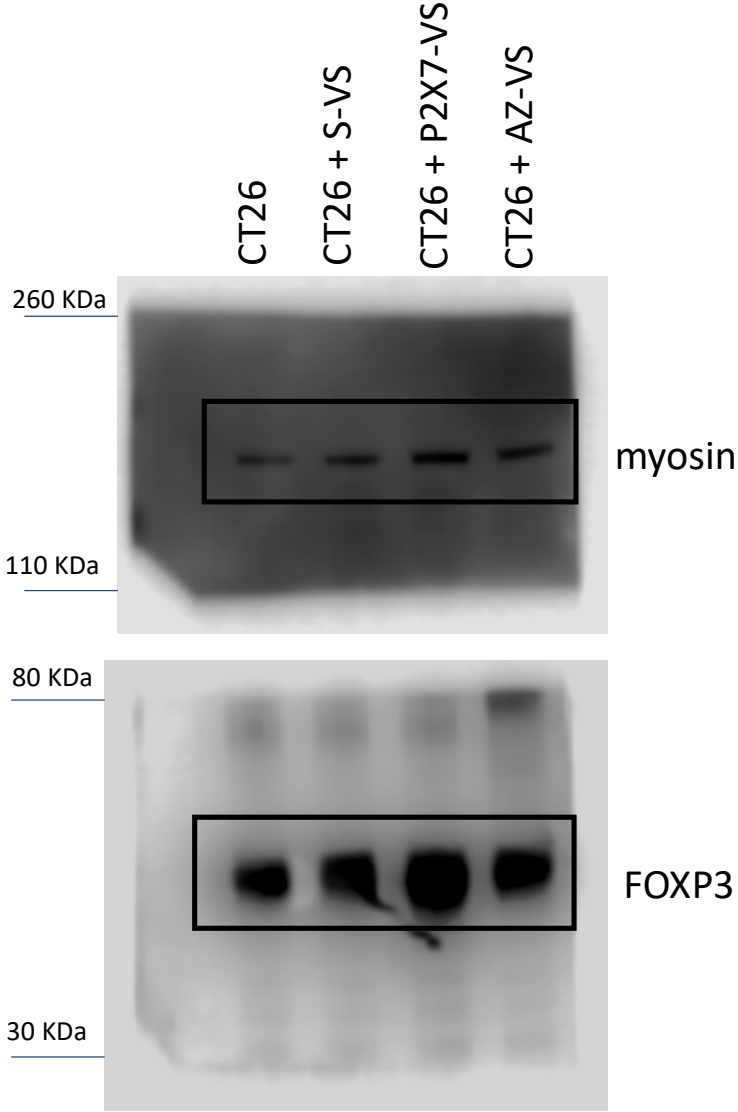

WB Suppl. figure 1

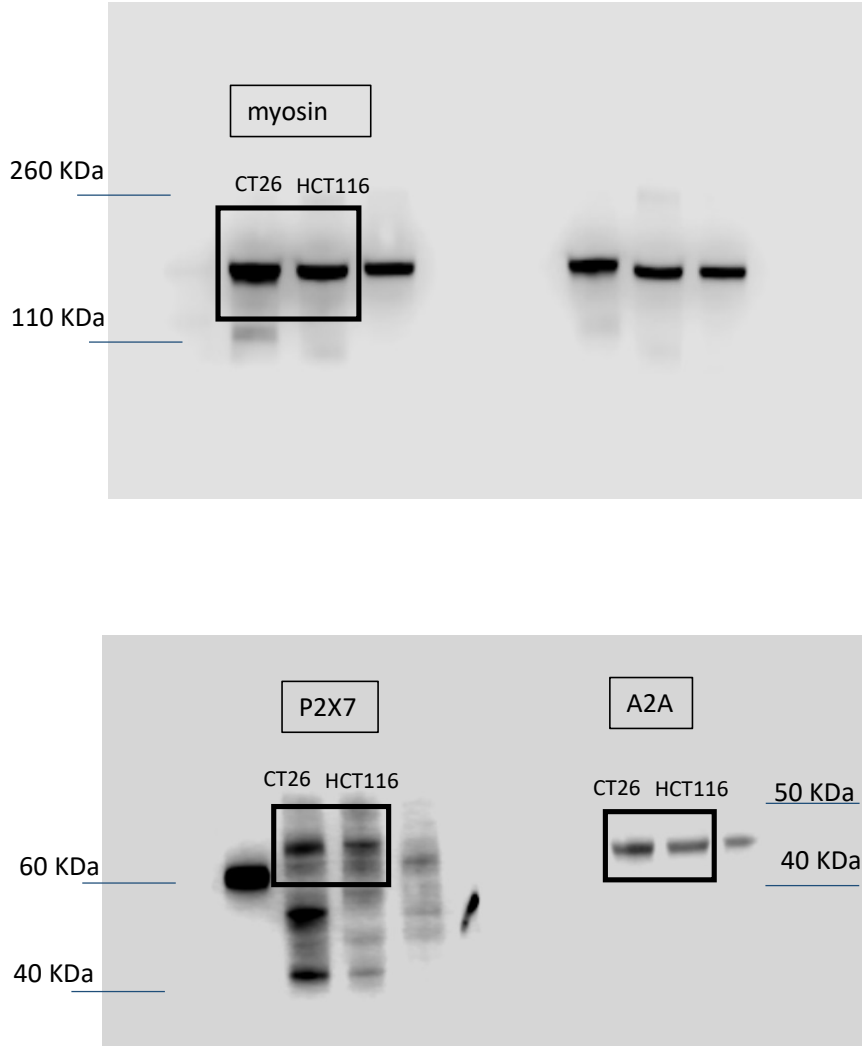

Supplement: Supplementary file 2 — Supplementary Figure 2 [file 41419_2025_7897_MOESM2_ESM.pdf]

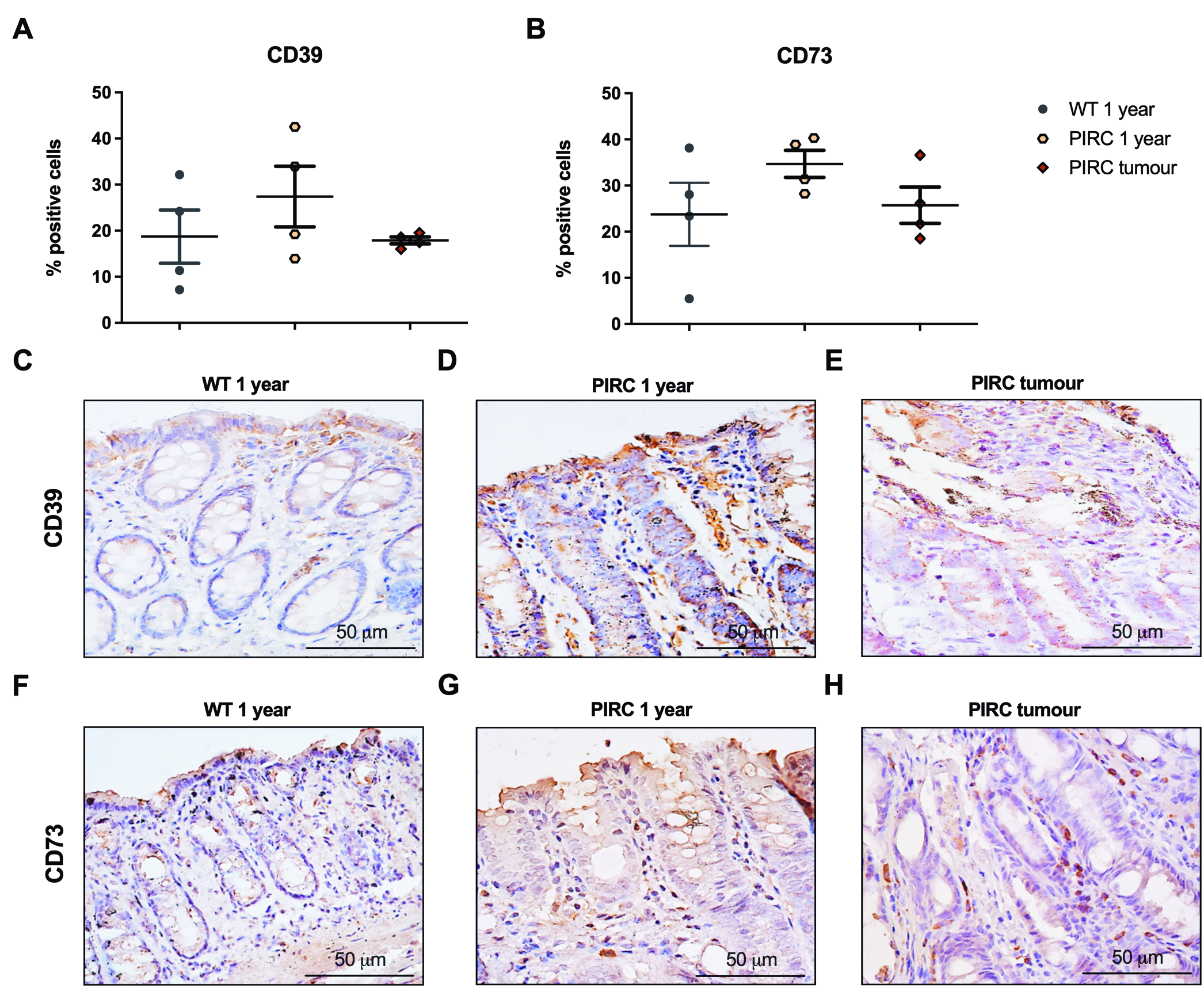

Supplement: Supplementary file 3 — Supplementary Figure 3 [file 41419_2025_7897_MOESM3_ESM.tif]
