# Supplementary material for: P2X7 a new therapeutic target to block vesicle-dependent metastasis in colon carcinoma: Role of the A2A/CD39/CD73 axis
Source: Cell Death Dis. 2025 Aug 4;16(1):587. doi: 10.1038/s41419-025-07897-2 (PMC12322077; doi:10.1038/s41419-025-07897-2)
Supplement: Supplementary file 4 — Supplemental material legends [file 41419_2025_7897_MOESM4_ESM.docx]

**Supplementary material legends.**

**Supplementary Fig.1. CT26 and HCT116 colon carcinoma cell lines express P2X7 and A2A**

(A) Western blot of myosin, P2X7, and A2A protein expression in the murine CT26 and human HCT116 colon carcinoma cell lines. Intracellular calcium rise following stimulation with 300 µM BzATP in CT26 (B) and HCT116 cells (D). Ethidium bromide uptake following stimulation with 300 µM BzATP in CT26(C) and HCT116 cells (E).

**Supplementary Fig.2. Uncropped Western Blots.** Full immunoblots corresponding to the panels shown in Fig.1 panel 1G, Fig.2 Panel H and Supplementary Fig.1 panel 1A.

**Supplementary Fig. 3. CD39 and CD73 protein expression doesn't change in WT and PIRC rats' colon.**

Percentage of positive cells for CD39 (A) and CD73 (B) in the colon of wt and PIRC rats at 1 year and in the PIRC's tumor (n=4 per condition). Representative images of immunohistochemistry staining for CD39 and CD73 in the colon of wt 1-year rat (C, F), and in the normal colon (D, G) and the tumor mass (E, H) of 1-year PIRC rat.

**Supplementary Video S1**

CT26 murine colon carcinoma cells were stimulated with the P2X7 agonist BzATP 300 µM. The cell membrane was stained with the red fluorescent dye PKH26. ATP and nucleic acids content was stained with the green fluorescent dye quinacrine. Total time course length 30 minutes.

**Supplementary Video S2**

HCT-116 human colon carcinoma cells were stimulated with the P2X7 agonist BzATP 300 µM. The cell membrane was stained with the FM4-64 red fluorescent dye . ATP and nucleic acids content was stained with the green fluorescent dye quinacrine. Total time course length 30 minutes.

**Supplementary Video S3**

CT26 murine colon carcinoma cells were incubated with P2X7 antagonist AZ10606120 (5 µM) for 10 minutes , corresponding to time 0 and subsequently stimulated with BzATP 300 µM. The cell membrane was stained with the red fluorescent dye PKH26. ATP and nucleic acids content was stained with the green fluorescent dye quinacrine. Total time course length 30 minutes.
