# Supplementary material for: P2X7 a new therapeutic target to block vesicle-dependent metastasis in colon carcinoma: Role of the A2A/CD39/CD73 axis
Source: Cell Death Dis. 2025 Aug 4;16(1):587. doi: 10.1038/s41419-025-07897-2 (PMC12322077; doi:10.1038/s41419-025-07897-2)
Supplement: Supplementary file 8 — Supplementary Table I [file 41419_2025_7897_MOESM8_ESM.docx]

**Table S1. Patient’s characteristics.** n/a not applicable. MUT mutated, WT wild-type.

| **PATIENT ID** | **GENDER** | **AGE** | **STAGE AT DIAGNOSIS** | **TUMOR LOCALIZATION** | **TNM AT DIAGNOSIS** | **SITES OF METASTASES** | **METASTASES**  **(Synchronous/Metachronous)** | **APC** |
| --- | --- | --- | --- | --- | --- | --- | --- | --- |
| **001** | F | 55 | III | Rectum | T4N1M0 | Lung, lymph nodes | M | MUT |
| **002** | M | 57 | IV | Right Colon | T4N2M1 | Liver, peritoneum | S | MUT |
| **003** | M | 59 | II | Left Colon | T3N0M0 | Liver, peritoneum | M | MUT |
| **004** | M | 66 | III | Right Colon | T3N2M0 | Liver, peritoneum | M | MUT |
| **005** | M | 70 | IV | Right Colon | T3N1M1 | Liver, peritoneum | S | WT |
| **006** | M | 84 | IV | Left Colon | T3N2M1 | Liver, peritoneum | S | WT |
| **007** | F | 50 | II | Right Colon | T4N0M0 | Lymphnodes, peritoneum | M | MUT |
| **008** | F | 83 | II | Right Colon | T3N0M1 | Lung | S | MUT |
| **009** | F | 62 | III | Right Colon | T2N1M0 | None | n/a | WT |
| **010** | F | 62 | III | Right Colon | T1N1M0 | Peritoneum | M | WT |
| **011** | M | 77 | IV | Right Colon | T4N1M1 | Liver | S | WT |
| **012** | F | 81 | III | Right Colon | T4N1M0 | Peritoneum | M | WT |
